# Precious Metal Dioxide Nanosheets: Bridging the Gap between Solution Chemistry and Solid-State Two-Dimensional Materials

**DOI:** 10.1021/acsmaterialsau.5c00183

**Published:** 2026-01-13

**Authors:** Satoshi Tominaka, Daisuke Takimoto, Akihiko Machida, Tomoya Eda, Yuki Nakahira, Yuki Tokura, Wataru Sugimoto

**Affiliations:** ⧫ Center for Basic Research on Materials (CBRM), National Institute for Materials Science (NIMS), 1-1 Namiki, Tsukuba, Ibaraki 305-0044, Japan; # Department of Chemistry, Biology and Marine Science, Faculty of Science, University of the Ryukyus, Nishihara, Okinawa 903-0213, Japan; § Graduate School of Medicine, Science and Technology, 13056 Shinshu University, 3-15-1 Tokida, Ueda, Nagano 386-8567, Japan; ¶ Institute for Aqua Regeneration, Shinshu University, 3-15-1 Tokida, Ueda, Nagano 386-8567, Japan; ‡ Synchrotron Radiation Research Center, National Institutes for Quantum Science and Technology (QST), Sayo, Hyogo 679-5148, Japan

**Keywords:** Oxide nanosheet, two-dimensional material, precious metal oxide, structural analysis, pair distribution function, chemical exfoliation, transition metal dichalcogenide

## Abstract

Unraveling the atomic structures of chemically exfoliated precious metal dioxide (PMD) nanosheets is the key to understanding their diverse properties and realizing their potential in applications like catalysis. Using pair distribution function (PDF) analysis, we have solved the structures of platinate and iridate nanosheets, revealing they both adopt a T-MoS_2_-type crystal structure. This discovery not only establishes a crucial structural analogy to well-understood transition metal dichalcogenides (TMDs) but, more importantly, allows us to explain the origins of their distinct properties. Our calculations based on these structures correctly predict that the platinate nanosheet is a yellow semiconductor, while the iridate nanosheet is a blue semimetal. Having established this powerful structure–property relationship, we further probed the unique chemical nature of these materials. We found that the structural polymorphism (T- vs T′-type) is governed by intrinsic elemental characteristics, rather than simple redox states as explored by in situ experiments. Instead of large-scale distortions, these nanosheets exhibit subtle short-range order (SRO) in their metal atom positions. This work provides a robust methodology for PMD research and highlights that chemically imparted features like SRO are key to designing the next generation of 2D materials.

## Graphical abstract

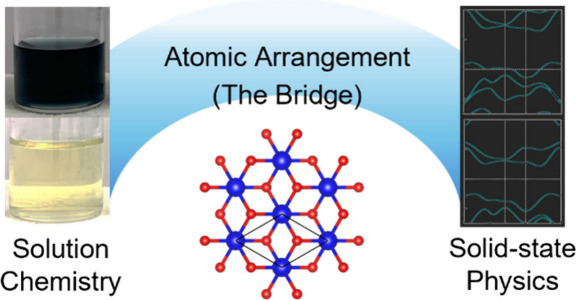

Chemically exfoliated precious metal dioxide (PMD) nanosheets are a compelling class of 2D materials, offering high atomic efficiency for applications like catalysis.^1,2^ However, unlike well-characterized crystalline materials such as transition metal dichalcogenides (TMDs),^3^ a lack of precise atomic-level structural knowledge has fundamentally limited the understanding and exploitation of their diverse electronic properties. Here we overcome this critical barrier for platinate^4^ and iridate nanosheets,^5^ materials of high interest for energy and sensing applications. By incorporating our prior structural elucidation of ruthenate nanosheets,^6^ this work establishes that the PMD family is a structural analogue to the renowned TMDs. Consequently, PMDs emerge as a compelling platform where the rigorous, physics-based understanding typical of TMDs^7^ can now be extended to 2D materials possessing the distinct advantages of chemical synthesis and solution-based processability.

The structural challenge in PMDs is exemplified by ruthenate nanosheets, for which conflicting reports on their electronic nature persisted for years.^8–10^ The ambiguity was resolved only when our group recently determined their definitive atomic arrangement as a T′-MoS_2_-type structure using pair distribution function (PDF) analysis,^6,11^ a powerful technique increasingly employed by researchers to elucidate the atomic structures of nanomaterials lacking long-range order, such as nanosheets.^11–13^ This breakthrough demonstrated that a detailed, TMD-like structural and electronic analysis is indeed possible for these chemically derived nanosheets, setting the stage for the work presented here.

Applying this proven PDF methodology, we now reveal that platinate and iridate nanosheets adopt T-MoS_2_-type structures, distinct from the distorted T′-type of ruthenate. Taken together, these findings establish a robust structural framework for the PMD nanosheet family as a whole, allowing for a comprehensive, TMD-like discussion of their physical properties for the first time. This is particularly significant as it unifies two distinct realms of 2D materials science. While much of TMD research focuses on materials on solid substrates for electronics, chemically exfoliated nanosheets exist as colloidal dispersions, making them uniquely suited for scalable applications in catalysis and energy storage. By providing the definitive atomic-level structures, our work enables the predictive power of solid-state physics to be harnessed for these chemically versatile nanomaterials, vastly expanding their potential for rational design.

Structural information on two-dimensional materials can be gained by PDF analysis^11^ (see Supporting Information for details on sample preparation and PDF data collection). We collected high-resolution PDFs using synchrotron high-energy X-ray diffraction, achieving a real-space resolution (Δ*r*) of approximately 0.23 Å. This resolution was calculated from a maximum scattering vector *Q*_max_ of 27 Å^–1^. This Δ*r* is generally sufficient for resolving the atomic arrangements of materials at room temperature. Initial investigations on both condensed solutions and dried powders of the platinate and iridate nanosheets revealed that the drying process induced restacking of the nanosheets, evidenced by the emergence of Bragg peaks corresponding to an interlayer spacing of 16.0 Å (Figure S1). The PDFs remained largely unchanged, indicating that the intralayer structure within individual nanosheets was preserved (Figures S2 and S3). Remarkably, the PDFs for the platinate and iridate nanosheets were nearly identical (Figure S4), despite their starkly different appearances, suggesting a common structural motif underlying these two materials.

The PDF of platinate nanosheet is shown in Figure 1a. The peak located around 2 Å is assigned to the nearest neighbor Pt–O distance, while the intense peak around 3 Å is assigned to the nearest Pt–Pt distance, judged by the Shannon radii and the X-ray atomic scattering factors.^14^ These features are different from the PDF of ruthenate (T′-MoS_2_, space group of *P*2_1_/m),^6^ where another peak assigned to the shorter Ru–Ru pairs appeared between these two peaks observed for platinate. To facilitate this comparison, a reanalysis of the ruthenate data, including the PDF fit and calculated partial PDFs, is provided in the Supporting Information (Figure S5). The interpretation of the X-ray PDF data indicates that the contribution of oxygen atoms is minor, and the fitting is successfully reproduced primarily by the influence of metal–metal (M–M) pairs and the contribution of metal–oxygen (M–O) pairs. Thus, the model validated the plausibility of the characteristic M–M pair distance well.

**Figure 1:**
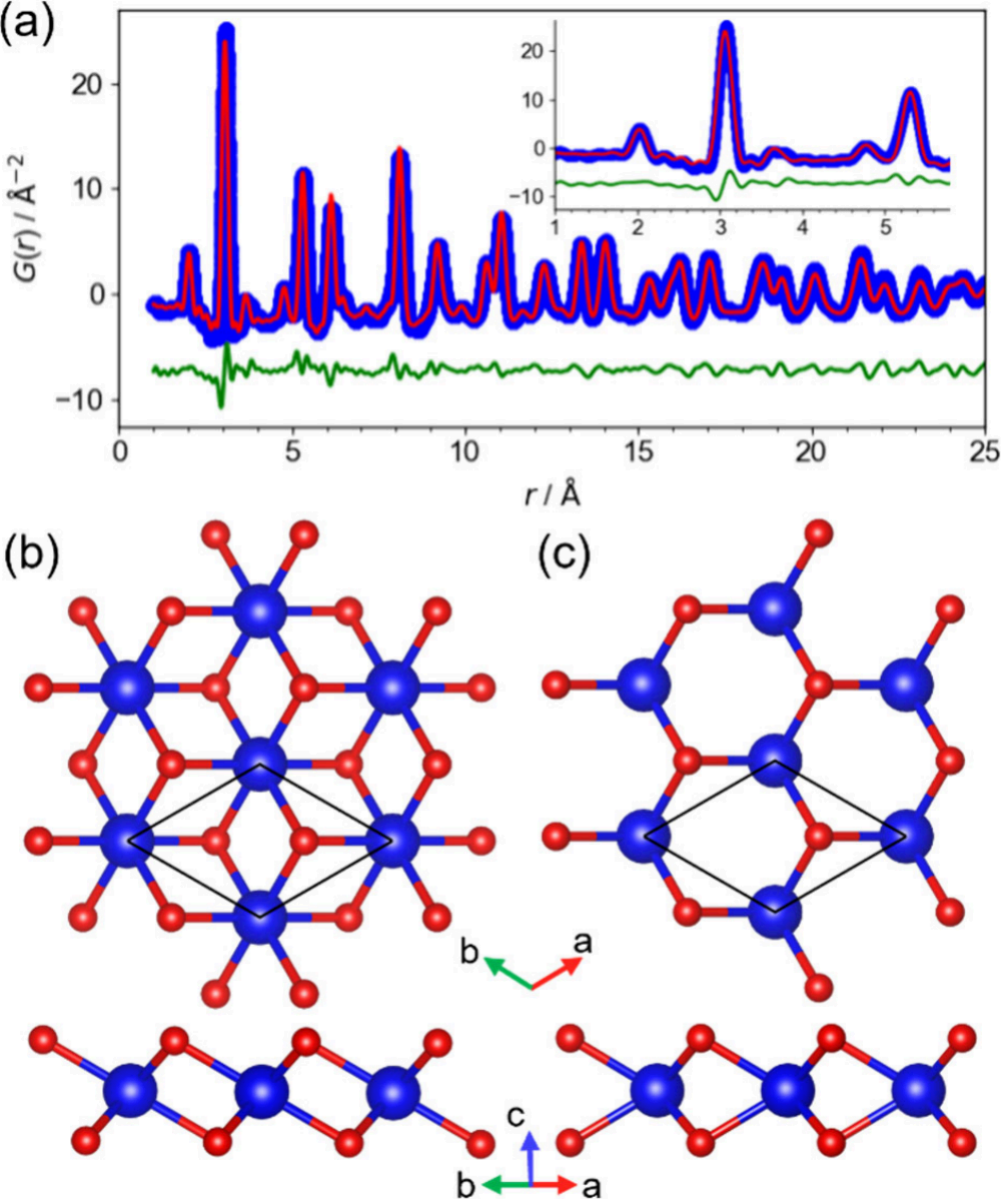
Pair distribution function (PDF) analysis and structural models of 2D platinate nanosheets. (a) Experimental PDF *G*(*r*) for platinate nanosheets (blue circles) with a fit (red solid line) using the T-type PtO_2_ model shown in (b). (b) Atomic structure of the T-type PtO_2_ model (trigonal, space group *P* 3 *m*1). The upper panel shows a view along the *c*-axis, and the lower panel shows a cross-sectional view. Solid lines depict the unit cell. (c) Atomic structure of the H-type PtO_2_ model (hexagonal, space group *P* 6 *m*2), similarly presented with top and cross-sectional views and unit cell indications. Comparison of these models highlights that the T-type and H-type structures differ solely in the positions of the oxygen atoms relative to the platinum layers.

The PDFs of both the platinate and iridate nanosheets were successfully simulated by adopting a T-MoS_2_ structural model (space group of *P* 3 *m*1, Figure 1a), where the metal atoms form a hexagonal arrangement. Because both the T-MoS_2_ model (Figure 1b) or H-MoS_2_ model (*P* 6 *m*2, Figure 1c) adopt hexagonal arrangements of metal atoms, the resulting electron diffraction pattern exhibits a typical 6-fold symmetry without diffuse streaks (Figure S6).^5^ This absence of streaks indicates the lack of significant in-plane defects, confirming a well-ordered hexagonal metal lattice. The structural similarity derived from this hexagonal arrangement is further reflected in the PDF fitting results, rendering them almost identical (Figures S7 and S8). It should be noted that the anion arrangement in these oxide nanosheets could not be definitively determined from X-ray PDF analysis due to the negligible contribution of O–O pairs to the total scattering signal compared to the dominant M–M and M–O pairs (Figure S7c,d).^15^ The partial PDFs show a physically unrealistic short nearest-neighbor O–O distance of ∼2 Å in the H-type structure (Figures S7d and S8d). This observation, combined with quantum chemical simulations indicating that the T-type structure is energetically more stable by 0.99 eV/atom for platinate (0.82 eV/atom for iridate), provides compelling evidence that the experimental samples adopt the T-type structure.

For the refinement using the T-MoS_2_ model, we employed a fitting range up to *r* = 25 Å. While the T-type average model accurately reproduces the long-range correlations (*r* > 10 Å), verifying the robust hexagonal framework, subtle discrepancies were observed in the short-range region. These local deviations suggest the presence of structural modulation, which will be discussed in detail as short-range order (SRO) in the latter part of this paper. Therefore, the 25 Å range was chosen to capture both the local variations and the average periodicity. The least-squares fitting revealed that the in-plane lattice parameter of platinate (*a* = 3.06578(4) Å) was slightly shorter than that of iridate (*a* = 3.10756(2) Å) (Figure S4). Considering that the Shannon radii, which represent the effective ionic radii in a crystal,^14^ of Pt and Ir are the same (0.625 Å), the difference may reflect differences in their electronic structure, or the degree of electron localization. This hexagonal lattice can simulate the reported two-dimensional X-ray diffraction pattern well (Table S1),^4^ confirming long-range structural order along the sheet structures.

With the T-type structures of platinate and iridate nanosheets established, and contrasted with the known T′-type ruthenate, we can now directly investigate how these structural differences manifest in their physical properties. A prime example is their distinct optical and electronic behavior. The elucidated atomic structures provide a direct basis for understanding the distinct optical properties of the nanosheets (Figure 2 and Figure S8). Visually, the colloids of the iridate, platinate, and ruthenate nanosheets appear deep blue, transparent yellow, and deep purple, respectively (Figure 2a). These colors are quantitatively reflected in their UV–vis absorption spectra (Figure 2b). As a reference, the semimetallic ruthenate nanosheet exhibits strong absorption across the entire measured range. In contrast, the iridate nanosheet displays a more complex profile; it has two distinct absorption regions in the long-wavelength range (>600 nm) and the ultraviolet range (<400 nm), with a valley of relative transparency around 500 nm, which accounts for its deep blue color. The pronounced absorption at long wavelengths is a hallmark of the intraband transitions expected in a semimetal. In further contrast, the platinate nanosheet is transparent at these long wavelengths, showing a clear absorption edge at higher energies, which is characteristic of a semiconductor.

**Figure 2:**
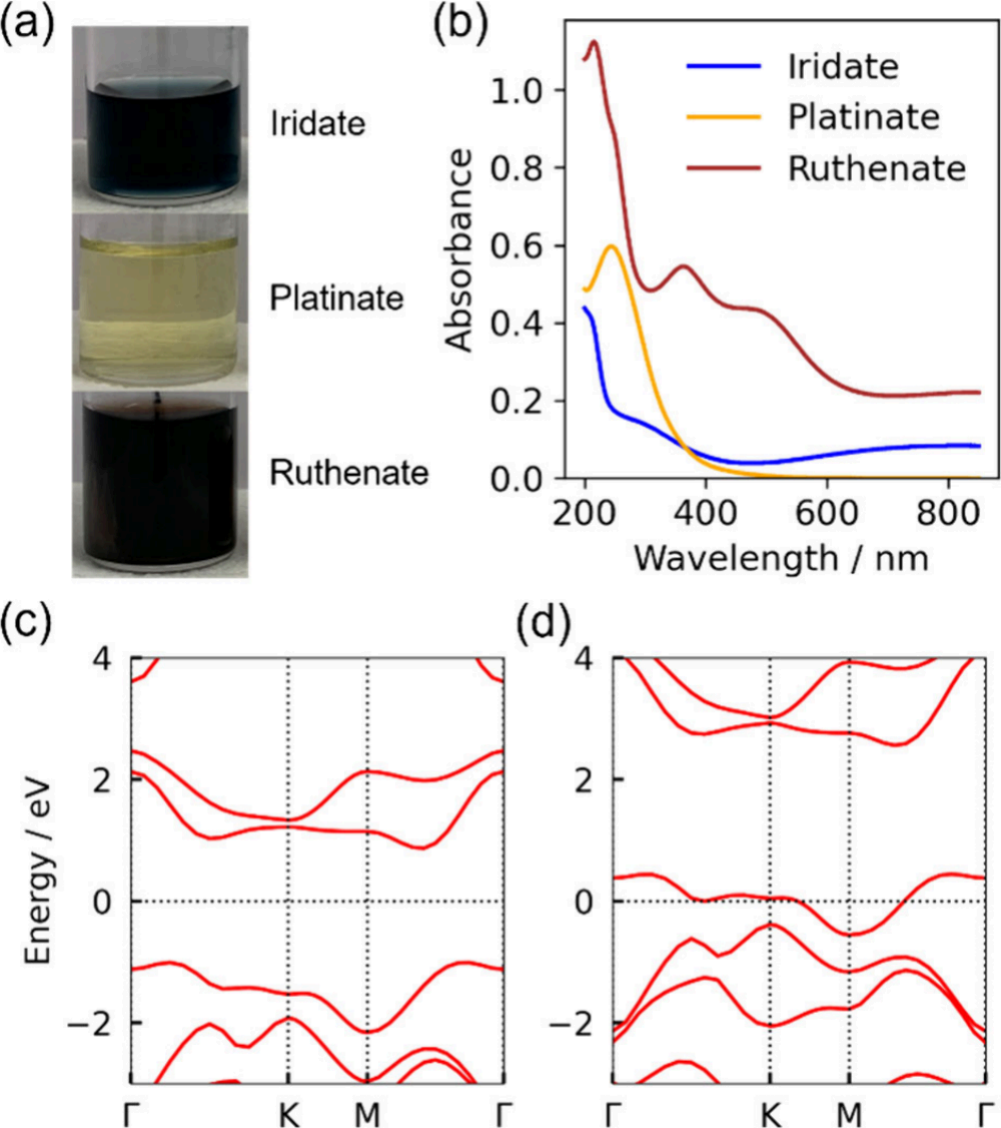
Optical properties and electronic structures of PMD-nanosheets. (a) Photographs of aqueous solutions (0.1 g L^–1^) of iridate, platinate, and ruthenate PMD-nanosheets, illustrating their distinct macroscopic colors. (b) UV–vis absorption spectra of the corresponding PMD-nanosheet solutions. (c) Calculated electronic band structure of T-type platinum dioxide, showing a band gap characteristic of a semiconductor. (d) Calculated electronic band structure of T-type iridium dioxide, demonstrating a band crossing the Fermi level, indicative of its semimetallic nature.

To uncover the electronic origins of the observed differences, we calculated band structures using density functional theory (Figure 2c and 2d). For these calculations, we employed a monolayer slab model with a 15 Å vacuum layer. This single-layer approximation is physically justified by the experimental stacking conditions; the presence of bulky tetrabutylammonium (TBA) cations and water molecules expands the interlayer distance (*d*-spacing) to ∼16 Å for both platinate and iridate nanosheets (Figure S1). At this large separation, direct electronic interactions between adjacent metal-oxide layers are negligible. Furthermore, in the colloidal dispersions where the distinct colors are observed, the nanosheets are solvated and effectively isolated. Thus, the vacuum-slab monolayer model appropriately captures the intrinsic electronic properties governing the optical behavior.

Note that, in addition to the energetical view, the band structure of the T-type models can account for the optical properties better than the H-type models (Figures S10 and S11). Thus, we mainly focus on the T-type model in the following discussion. Our first-principles band structure calculations for the T-type model of platinum dioxide reveal a clear semiconducting nature, with an indirect band gap of ∼2 eV and a larger direct gap of ∼3.5–4 eV (Figure 2c, Figure S10). This theoretical result aligns perfectly with its experimental properties (Figure S9); the transparent yellow color of the solution arises from an absorption edge corresponding to this band gap, while the transparency at lower energies confirms the absence of the intraband transitions characteristic of a metal. This excellent agreement for the platinate system strongly validates our structural model.

The case of the iridate nanosheet is more nuanced. Our calculation for the ideal, isolated T-type monolayer predicts a metallic state due to a band crossing the Fermi level (Figure 2d, Figure S11). The presence of multiple low-energy electronic excitations in this model, such as an intraband transition calculated to be around ∼2 eV, provides a qualitative basis for understanding the strong optical absorption observed at long wavelengths, which gives rise to the deep blue color. However, it must be noted that this calculation represents an ideal, intrinsic state. Recent transport measurements on real-world monolayer films have shown semiconducting (thermally activated) behavior,^16^ likely due to carrier depletion by surface states or environmental interactions. This is consistent with observations that thicker, multilayer films of the same nanosheets behave metallically, suggesting the inner layers retain the intrinsic character predicted here. Thus, our model captures the intrinsic electronic nature of the T-type IrO_2_ layer, while the observed properties highlight the critical role of surface effects.

With the electronic structures of the T-type polymorphs understood, we can definitively rule out the alternative H-type structure. Our calculations predict that the hypothetical H-phases of both platinate and iridate would be metallic (Figures S10b and S11b). This is fundamentally inconsistent with the established wide-gap semiconducting nature of platinate and the observed semiconducting transport of the iridate monolayer. Thus, the consistency across all three material systems, including ruthenate (Figures S5 and S12), provides a final, compelling confirmation of our structural assignments based on a powerful combination of structural, energetic, and electronic evidence.

Having validated our structural models against their electronic properties, we now turn to the nuanced chemical state that distinguishes these exfoliated nanosheets. A fundamental difference from TMDs lies in the chemical nature imparted by the exfoliation process.^2,17^ This process inherently yields anionic nanosheets with an excess negative charge, implying that the metal sites exist in a nonstoichiometric or mixed-valence state such as Pt^4+^/Pt^3+^, Ir^4+^/Ir^3+^ and Ru^4+^/Ru^3+^. We hypothesize that this chemically induced redox state is the primary driver for stabilizing specific polymorphs, such as the T′-type structure in ruthenate. Given that the Ru^3+^ oxidation state is known to be particularly stable, we initially suspected that a greater proportion of the Ru^3+^ state—meaning a higher overall electron density in ruthenate relative to iridate and platinate—would favor the T′-transition.

The mixed valence or ionic states offers a stark contrast to the mechanism in charge-neutral TMDs, where such periodic lattice distortions are almost exclusively attributed to intrinsic electronic instabilities like charge density waves (CDWs).^7^ This chemical sensitivity provides a compelling explanation for existing ambiguities in the literature; for instance, different studies on ruthenate nanosheets have reported various polymorphs (e.g., T′, T, or H-type), even when employing ostensibly similar synthetic methodologies. This variability strongly suggests that subtle, often overlooked chemical differences—such as the nature of interlayer counterions or the degree of surface protonation—play a decisive role in stabilizing a specific structure.

This hypothesis—that the T′ structure is a manifestation of a specific redox state—prompts a critical test: can we induce a T-to-T′ transformation by chemically tuning this state? To explore this directly, we performed in situ PDF measurements on the T-type platinate nanosheets under a reducing environment. The time-resolved data (Figure 3, Figures S13–S15) clearly illustrates a direct conversion from the initial oxide structure to a face-centered cubic (FCC) metal. To rigorously search for a T′-like intermediate, we generated a reference PDF pattern for a hypothetical T′-platinate structure (Figure 3c). This model was created by substituting Pt atoms into our newly refined structure of T′-ruthenate (Figure S5), providing a chemically reasonable target for what a distorted intermediate phase would look like. A comparison with this reference pattern confirms that the characteristic peaks of the T′-phase are absent in our experimental data, indicating that no such intermediate is formed. This result suggests that the T′-structure of ruthenate is not a universally accessible, slightly reduced state of a generic T-type PMD. Instead, the structural preference likely stems from more intrinsic elemental characteristics. Nevertheless, this experiment powerfully demonstrates the utility of in situ PDF in tracking the dynamic reactivity and decomposition pathways of PMD nanosheets, which is essential for their rational design.

**Figure 3:**
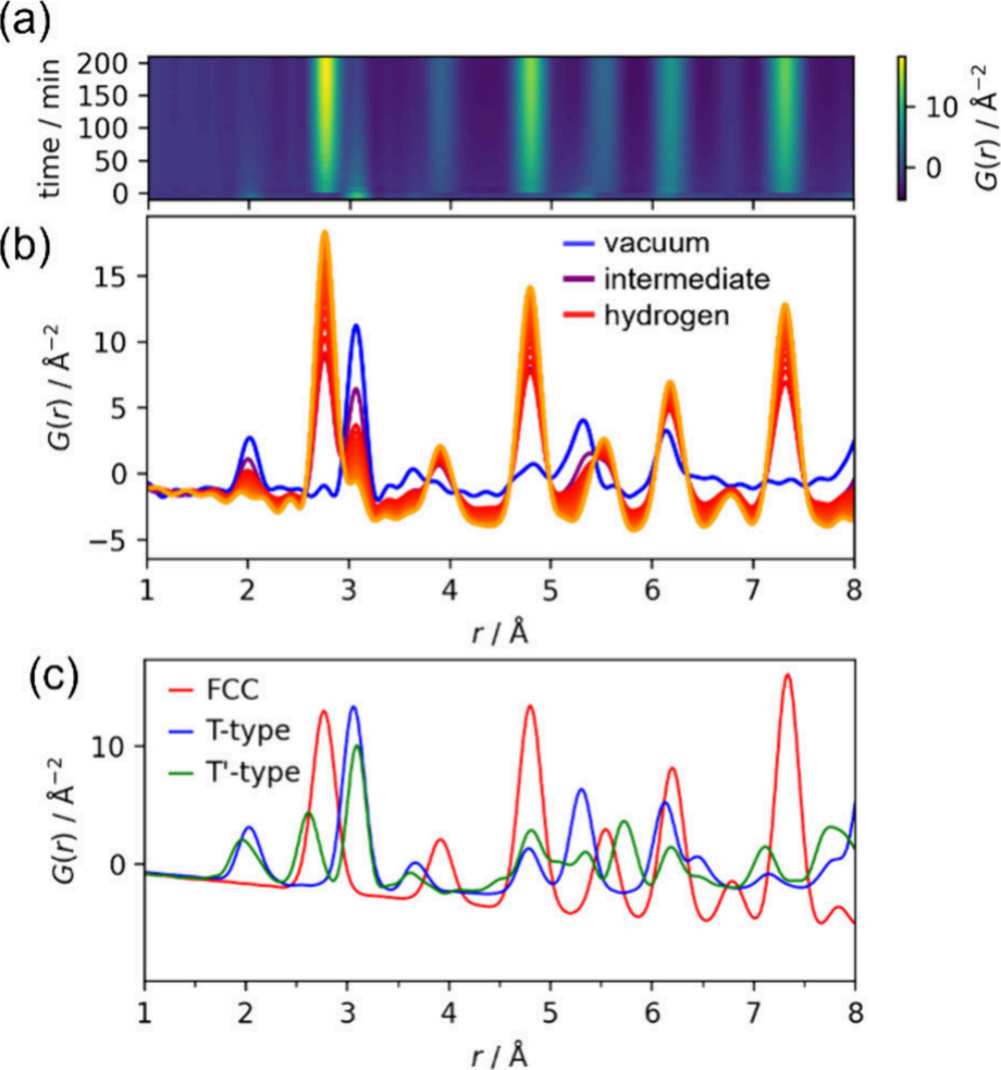
In-situ pair distribution function (PDF) analysis tracking the structural changes of platinate nanosheets upon reduction. (a) Time-resolved 2D contour plot of *G*(*r*). Hydrogen gas was introduced at *t* = 0, initiating the direct conversion from the initial oxide structure to a metallic phase. The color scale represents PDF intensity. Each frame represents a 10 min data acquisition. (b) Selected PDF patterns *G*(*r*): platinate nanosheets under vacuum (blue curve); the 10 min data acquisition capturing the transition from vacuum to H_2_ atmosphere (purple curve); and representative subsequent patterns under H_2_ atmosphere (red curves, progressing to lighter orange hues over time). (c) Calculated reference PDFs for the initial T-type oxide, the final FCC metal phase, and a hypothetical T′-type intermediate. The T′-platinate model was generated by substituting Pt atoms into the experimentally refined structure of T′-ruthenate (see Figure S5). The absence of characteristic T′-phase peaks (e.g., a short Pt–Pt distance around 2.6 Å) in the experimental data supports a direct conversion pathway.

The appearance of the T′-structure uniquely in ruthenate is fundamentally dictated by the valence electron count and the resulting occupation state of the e_*g*_ orbitals within the octahedral crystal field. Experimental data, such as the direct reduction of T-platinate to Pt metal without forming an intermediate T′-phase, proves that simply inducing a mixed M^3+^/M^4+^ valence state is insufficient to trigger the structural transition. Instead, the instability is attributed to the low electronic population inherent to Ru. While Pt and Ir possess a sufficient number of e_*g*_ electrons (approaching half-filling or greater) to maintain the original T-phase symmetry and stability, Ru exhibits the lowest e_*g*_ occupancy (i.e., the most significant hole density). This critical electronic deficit generates a robust thermodynamic driving force for the system to gain energy through the formation of new Ru–Ru bonds. This spontaneous d–d coupling causes a subsequent distortion of the Ru sublattice, culminating in the formation of interconnected Ru–Ru bonding networks that define the T′-phase.

This study represents a significant step forward in the materials science of PMD nanosheets. By combining PDF analysis with first-principles calculations, we have successfully determined the precise atomic structures of platinate and iridate nanosheets. This structural knowledge, a long-standing challenge in the field, enabled us to directly explain their distinct electronic and optical properties, elevating the study of PMDs to a level of detail comparable to that of mature fields like TMDs.

Our findings also highlight the unique chemical nature of these materials, evident in their local atomic structure. While our average T-type model accurately captures the primary structural features, detailed PDF analysis reveals that the nearest metal–metal (M–M) distances at ∼3 Å are consistently longer than those derived from the average unit cell (Figure S3). More specifically, the discrepancy between the observed M–M distances and the simulated T-type model is not constant but varies with distance (Figure S16a). By assuming an out-of-plane displacement of metal ions (buckling), we found that the vertical offsets between M–M pairs are broadly distributed around ∼0.3 Å, ranging from a minimum of ∼0.0 Å to a maximum of ∼0.6 Å (Figure S16b). This continuous variation implies a sinusoidal nature of the buckling rather than discrete steps. Significantly, the largest offset (∼0.6 Å) corresponds to an M–M separation of ∼9.2 Å—three times the unit cell *a*-axis. Interpreting this distance as the half-period (peak-to-valley separation) of the wave implies a full modulation periodicity of six unit cells.

Accordingly, we modeled this commensurate modulation using a sinusoidal wave within a 6 × 6 supercell (Figure 4 and Figure S16c and eq 2.14 in the Supporting Information). This model successfully reproduces the experimental PDF data (Figure 4a); notably, the goodness of fit around the nearest-neighbor M–M distance (∼3 Å) is significantly improved compared to the model without modulation (Figure 4b). It is important to note that this modulation introduces purely out-of-plane buckling with an optimized amplitude of 0.51 Å (Figure 4c), while fully preserving the in-plane hexagonal metal arrangement (Figure 4d). This chemical short-range order (SRO)—manifesting as commensurate buckling—likely reflects charge localization and serves as a structural fingerprint of the chemical exfoliation process, distinguishing these nanosheets from their perfectly crystalline counterparts.

**Figure 4:**
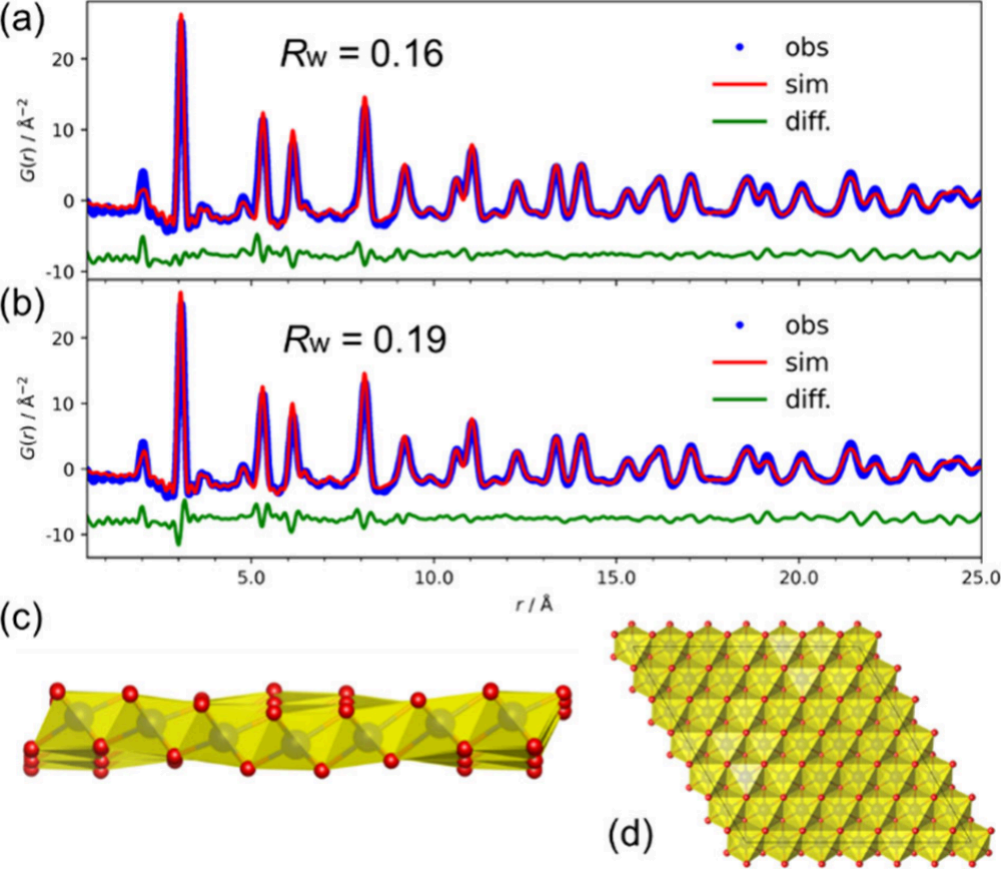
Analysis of short-range order using the commensurate sinusoidal modulation model. (a, b) Experimental *G*(*r*) for platinate nanosheets (blue circles) fitted with the calculated one (red solid lines) based on a 6 × 6 supercell of the T-type PtO_2_ structure. The fit in (a) incorporates the sinusoidal vertical modulation, whereas the fit in (b) assumes a flat layer without modulation. In the modulated model (a), oxygen coordinates were locally optimized within a range of ± 0.01 fractional coordinates to accommodate the buckling. (c, d) Visualizations of the commensurate modulated structural model. (c) Side view illustrating the sinusoidal buckling propagating along the *b*-axis. (d) Top view showing that the hexagonal arrangement of metal ions is fully preserved.

How this inherent structural disorder precisely impacts the electronic properties presents an exciting avenue for future research. Notably, the commensurate buckling revealed here offers a compelling explanation for the puzzling semiconducting behavior of the iridate nanosheets, which are predicted to be metallic in the flat limit. The influence is 2-fold: fundamentally, the disruption of perfect translational symmetry and the creation of a superlattice potential can induce charge localization, opening a band gap at the Fermi level. Locally, the resulting distribution of M–M distances and bond angles directly modulates orbital overlap, affecting bandwidth and local electronic states. This combination could be the key to unlocking novel electronic or catalytic functionalities not found in ideal 2D lattices. Fully exploring this nexus between their chemical synthesis, short-range structure, and detailed physical property measurements is a crucial next step in the development of this compelling class of materials.

These insights open exciting avenues for future research. By leveraging the vast knowledge from the TMD field as a guide, while simultaneously focusing on the unique opportunities presented by chemical exfoliation—such as controlling SRO or polymorph stability through surface chemistry—PMD nanosheets offer a fertile ground for discovering novel functionalities. Specifically, the discovery of commensurate buckling induced by interlayer interactions suggests that the electronic structure of these materials is highly sensitive to the local electrostatic environment. This structural flexibility makes them ideal candidates for chemiresistive sensors, where surface adsorption could modulate the buckling amplitude and drastically alter conductivity, potentially exceeding the sensitivity of rigid oxide counterparts. Furthermore, particularly for iridates, the interplay between strong spin–orbit coupling and the charge localization derived from this SRO points toward unexplored territories in correlated electron physics, offering potential applications in quantum information technology or spintronics as novel Mott insulators or topological materials. This deeper, structure-based understanding paves the way for the rational design of these high-performance 2D materials for such targeted applications.

## Supplementary Material



## Notes

Experimental details, X-ray total scattering, quantum chemistry, and detailed method for PDF analysis (PDF)

## †

Graduate School of Advanced Science and Engineering, Hiroshima University, 1-7-1 Kagamiyama, Higashihiroshima, Hiroshima 739-8521, Japan
