# Supplementary material for: Precious Metal Dioxide Nanosheets: Bridging the Gap between Solution Chemistry and Solid-State Two-Dimensional Materials
Source: ACS Mater Au. 2026 Jan 13;6(2):313–8. doi: 10.1021/acsmaterialsau.5c00183 (PMC12983095; doi:10.1021/acsmaterialsau.5c00183)
Supplement: Supplementary file 1 [file mg5c00183_si_001.pdf]

Supporting information file

Precious Metal Dioxide Nanosheets: Bridging the Gap between Solution Chemistry and Solid-State Two-Dimensional Materials

Satoshi Tominaka [♦,*], Daisuke Takimoto[#], Akihiko Machida[‡], Tomoya Eda[§], Yuki Nakahira[‡,†], Yuki Tokura[¶], Wataru Sugimoto[§,¶]

[♦]Center for Basic Research on Materials (CBRM), National Institute for Materials Science (NIMS), 1-1 Namiki, Tsukuba, Ibaraki 305-0044, Japan.

[#]Department of Chemistry, Biology and Marine Science, Faculty of Science, University of the Ryukyus, Nishihara, Okinawa 903-0213, Japan.

[§]Graduate School of Medicine, Science and Technology, Shinshu University, 3-15-1 Tokida, Ueda, Nagano 386-8567, Japan.

[¶]Institute for Aqua Regeneration, Shinshu University, 3-15-1 Tokida, Ueda, Nagano 386-8567, Japan.

[‡]Synchrotron Radiation Research Center, National Institutes for Quantum Science and Technology (QST), SPring-8, Sayo, Hyogo 679-5148, Japan

*Corresponding author (email): tominaka.satoshi@nims.go.jp

†Present address: Graduate School of Advanced Science and Engineering, Hiroshima University, 1-7-1 Kagamiyama, Higashihiroshima, Hiroshima 739-8521, Japan.

Experimental details

S1. Synthesis of PMD nanosheets

Platinate nanosheets: The potassium platinate was synthesized by the calcination of a pelletized mixture of PtO_2 (Combi-Blocks, Inc.) and K_2CO_3 (1:1 molar ratio) at 800°C for 1 h in air. The resultant compounds were washed using ultra-pure water to remove water soluble components. This compound was protonated by acid treatment using aqueous 1 M HCl at room temperature for 3 days. The aqueous HCl was replaced daily. The protonated platinate was exfoliated into colloidal platinate nanosheets by reaction with aqueous tetrabutylammonium hydroxide (TBAOH, FUJIFILM Wako Pure Chemical Industries) for 10 days. The resultant suspension was centrifugated at 4000 rpm for 30 min to remove the unexfoliated compounds. Note that the nanosheets samples prepared by two different laboratories (Ryukyu University and Shinshu University) exhibit identical PDFs and the process is confirmed reproducible well.

Iridate nanosheets: The potassium iridate was synthesized by the calcination of a pelletized mixture of IrO_2 (FUJIFILM Wako Pure Chemical Industries) and K_2CO_3 (1:1 molar ratio). The pelletized mixture was first calcinated at 750°C for 1 h under Ar flow. The resultant compounds were mixed and pelletized again, and calcinated at 780°C for 1 h under Ar flow. The calcination was repeated three times. The resultant compounds were washed using ultra-pure water to remove water soluble components. This compound was protonated by acid treatment using aqueous 1 M HCl at 60°C for 3 days. The aqueous HCl was replaced daily. The protonated iridate was exfoliated into colloidal iridate nanosheets in aqueous TBAOH for a week. The resultant suspension was centrifugated at 2000 rpm for 30 min to remove the unexfoliated compounds.

Ruthenate nanosheets. Ruthenate nanosheets were synthesized based on the previous reports. The layered potassium ruthenate was synthesized by the calcination of a pelletized mixture of RuO_2 (FUJIFILM Wako Pure Chemical Industries) and K_2CO_3 (8:5 molar ratio) at 850°C for 12 h under Ar flow. The resultant compounds were washed using ultra-pure water to remove water soluble components. This compound was protonated by acid treatment using aqueous 1 M HCl at 60°C for 3 days. The aqueous HCl was replaced daily. The protonated ruthenate was added to aqueous TBAOH and subsequently shaken for 10 days for the exfoliation into colloidal ruthenate nanosheets. The resultant suspension was centrifugated at 2000 rpm for 30 min to remove the unexfoliated compounds.

S2. Samples for PDF measurements

To accurately determine the structures of the exfoliated nanosheets, it is essential to assess the structural consistency between samples prepared by different procedures. This is of practical importance because precious metal dioxides are likely to be reduced and/or the structural transformation might occur. Therefore, we conducted structural investigations on both condensed solutions (solvated sample) and dried powders. The powder samples were placed in 1 mmϕ Kapton capillaries (Cole-Parmer).

1) Procedure 1: The aqueous solvent of nanosheet dispersion was concentrated by centrifugation at 3500 rpm for a few days.
2) Procedure 2: The solvent of the sample prepared by the procedure 1 was replaced with ethanol a few times, and then vacuum-dried overnight. This sample was slightly sticky.
3) Procedure 3: The aqueous solvent of nanosheet dispersion was replaced with acetone a few times, centrifuged at 3500 rpm, and then vacuum-dried overnight. This sample was not sticky.

The sample prepared by Procedure 2 introduced Bragg peaks in the low-angle region of the diffraction patterns, confirming the formation of ordered structures through nanosheet restacking. However, the PDFs remained largely unchanged, with only minor variations observed. This suggests that while drying induces periodicity along the stacking direction, the intralayer structure within individual nanosheets remains consistent.

To further address potential complications arising from excess tetrabutylammonium cations, we employed a solvent exchange procedure, replacing the original solvent with acetone and thoroughly rinsing the material. Acetone was chosen for its ability to effectively solvate the nanosheets while also being easily removed during drying. This process served two crucial purposes: it minimized the contribution of extraneous tetrabutylammonium cations and associated solvent to the scattering signal, and it facilitated the formation of a fine, dry powder suitable for PDF measurements. The resulting dried powder exhibited PDFs consistent with the preliminary data obtained from the solvated samples and dried powders.

S3. PDF measurements.

X-ray total scattering data for PDF analysis were collected at beamline BL22XU at SPring-8 using synchrotron radiation. The X-ray wavelength was λ = 0.181050 Å, calibrated using a CeO_2 standard (a = 5.41165 Å). Data were acquired using a Varex Imaging XRD1621 flat panel detector. Instrumental parameters required for the conversion of 2D scattering data into 1D intensity profiles, such as the sample-to-detector distance (determined to be 225.7864 mm), were calibrated using a CeO_2 standard, while peak broadening functions used for PDF simulation were determined using a Ni standard. For ex situ PDF measurements, powdered samples were loaded into polyimide capillaries with an outer diameter (OD) of 1.1 mm (Cole-Parmer). For each PDF pattern, 0.1-second exposures were typically collected and integrated for a total of 1 minute.

In-situ PDF measurements during hydrogen reduction were conducted using 1.59 mm OD polyimide capillaries (MicroLumen). Each capillary was connected to a gas handling system via Swagelok fittings, enabling evacuation and controlled gas introduction. Prior to H_2 exposure, the sample capillary was typically evacuated to a gauge pressure of −0.096 MPa. Subsequently, hydrogen gas was introduced to achieve the target pressures detailed in the respective figure captions (e.g., 0.0 MPa or −0.092 MPa gauge). For each PDF pattern, 8-second exposures were typically collected and integrated for a total of 10 minutes. To confirm that the observed structural transformations were primarily driven by chemical reduction with hydrogen and not by X-ray beam-induced effects, a control experiment was performed. After an in-situ reduction experiment where a specific region of the sample was continuously exposed to both H_2 gas and the X-ray beam, the sample capillary was evacuated. The sample stage was then translated to an adjacent region of the nanosheet sample within the capillary. This region had been exposed to the same H_2 gas environment for the same duration but had not been irradiated by the X-ray beam. Subsequent PDF measurement of this previously un-irradiated region (under vacuum) revealed a similar extent of reduction to that observed in the continuously irradiated region, confirming that the structural changes were predominantly caused by the chemical interaction with hydrogen gas.

From the two-dimensional raw intensity data of the sample, only the intensities of the capillary were subtracted as background intensity (I_{bg}). Polarization, oblique incidence with absorption correction for the CsI scintillator layer, and area corrections were performed. The data was then converted into 1D total scattering data using the PIXIA program.[1] For treating solvated nanosheet materials, 1D total scattering data for the supernatant collected

in the centrifuge process was subtracted from the intensity of the solvated sample with reducing the PDF peaks assigned to the solvent. After correcting fluorescence intensity (I_{flu}) and Compton scattering intensity (I_{com}), the data reflects coherent scattering intensity, I_{coh}. Then the I_{coh} data was normalized with the form factor based on the Faber-Ziman formalism calculated using atomic scattering factors (f) with the MaterialsPDF program[1] to be the structure-function, $S(Q)$.

$$S(Q) = \frac{I_{coh} - \{\langle f(Q)^2 \rangle - \langle f(Q) \rangle^2\}}{\langle f(Q) \rangle^2}$$

where $\langle f(Q)^2 \rangle - \langle f(Q) \rangle^2$ is the Laue monotonic scattering term, N is the number of scattering atoms, and $f(Q)$ is the atomic scattering factor. $S(Q)$ was converted into reduced PDFs, $G(r)$, by Fourier transforms in the Q range of 1.0–28.0 Å^{-1}.

$$G(r) = \frac{2}{\pi} \int_{Q_{min}}^{Q_{max}} Q(S(Q) - 1) sin(Qr) dQ$$

The resulting experimental $G(r)$ was subsequently analyzed by fitting structural models, as described in the following section.

S4. PDF simulation

The PDF data for the isolated nanosheet model were calculated using an in-house program written in Python to simulate the structural models with periodic boundary conditions along the nanosheet as reported previously.[2] The calculations were based on the following equations using vectors and written to be processed by the SciPy and NumPy modules of Python.[3] The PDF is calculated for atomic pairs at a distance r_{ij}:

$$r_{ij} = \left| \overrightarrow{r_{ij}} + \vec{t} \right| \quad (2.1),$$

where $\overrightarrow{r_{ij}}$ is the vector from atom i to atom j within the unit cell and $\vec{t}$ is the translation vector for extending the structure defined by the unit cell. The translational vectors along the nanosheet are obtained by calculating the periodic boundary conditions along the in-plane directions. The amplitude of scattering of these pairs was calculated as follows:

$$g_{ij}^{0} = \frac{c_i c_j \cdot f_i f_j}{\langle f \rangle^2} \quad (2.2),$$

$$\langle f \rangle = \frac{\sum_i c_i f_i}{\sum_i c_i} \quad (2.3),$$

where c_i and f_i are the concentration and scattering factor of species *i* in the unit cell, respectively. These discrete data are broadened by atomic motion in real materials using atomic displacement parameters, as used in crystallography, where Gaussian broadening is assumed.

$$A(r) = \frac{1}{\sqrt{2\pi}\sigma_{ij}} \exp\left(-\frac{r^2}{2\sigma_{ij}{}^2}\right) \quad (2.4),$$

$$\sigma_{ij} = \sqrt{U_{\mathrm{iso},i} + U_{\mathrm{iso},j}} \quad (2.5),$$

where $A(r)$ is a Gaussian function applied by convolution that simulates PDF peak broadening and σ_{ij} is the deviation of the peak for the pairs of *i* and *j* calculated using the atomic displacement parameter U_{iso}.

The PDF simulation for non-bulk materials was simulated by taking the influence of a limited Q range, in particular, the influence of limited Q minimum as done previously.[4,5]

$$G_r^{\mathrm{o}} = \frac{1}{r \cdot n_p}\left(\sum_{i,j}\left(g_{ij}^{\max} * A(r)\right) - \sum_{i,j}\left(g_{ij}^{\min} * A(r)\right)\right) \quad (2.8),$$

$$g_{ij}^{\max} = c_i c_j \cdot \int_0^{Q_{\max}} \frac{f_i(Q) \cdot f_j(Q)}{\langle f \rangle^2} dQ \quad (2.9),$$

$$g_{ij}^{\min} = c_i c_j \cdot \int_0^{Q_{\min}} \frac{f_i(Q) \cdot f_j(Q)}{\langle f \rangle^2} dQ \quad (2.10),$$

where $g_{ij}^{\max}$ and $g_{ij}^{\min}$ are the cosine transforms (Fourier transforms) of Q-dependent atomic scattering factors. To reduce the computational cost, the $g_{ij}^{\min}$ was approximated as follows:

$$G_r^{\mathrm{o}} = \frac{1}{r \cdot n_p}\left(\sum_{i,j}\left(g_{ij}^{\max} * A(r)\right) - \sum_{i,j}\left(g_{ij}^{\mathrm{z}} * A(r)\right) * s\right) \quad (2.11),$$

$$g_{ij}^{z} = c_i c_j \cdot \frac{z_i z_j}{\langle z \rangle^2} \quad (2.12),$$

$$s = \mathrm{sinc}\left(\frac{r}{\pi \cdot Q_{\min}}\right) \quad (2.13),$$

where g_{ij}^{z} is the scattering amplitude for the atomic pairs *i* and *j* based on their electron numbers z_i and z_j, and *s* is a sinc function associated with the Fourier transform between 0 and $Q_{\min}$.

The experimentally obtained G(r) was analyzed by the curve fitting procedure using the least-squares minimization (scipy.optimize.least_squares function with the Trust Region Reflective algorithm and a linear loss function under the lmfit interface written in python).

We modeled the commensurate sinusoidal modulation along the *a*- and *b*-axes as an out-of-plane displacement (Δz) using the following equation:

$$\Delta z(x, y) = A \cos(2\pi\, x) \cos\,(2\pi\, y) \quad (2.14),$$

where A represents the amplitude of the buckling, and x and y denote the fractional coordinates within the supercell.

S5. Quantum chemical calculation.

The electronic properties of the nanosheet material were investigated using density functional theory (DFT) as implemented in the Quantum ESPRESSO software package (version 7.4). We employed the projector augmented wave (PAW) method to describe the interaction between the core and valence electrons. This method offers an accurate and efficient way to represent the electronic structure of materials. To account for relativistic effects, scalar-relativistic pseudopotentials were employed throughout the calculations.

Structural Relaxation: To simulate the isolated nanosheet, a vacuum layer of 15 Å was introduced in the direction perpendicular to the nanosheet plane, resulting in a total thickness of 18.5 Å in the simulation cell. Our calculations began with a structural model derived from experimental data. To ensure accuracy, we first refined this initial structure by allowing the oxygen atoms to adjust their positions within the material, while keeping the overall dimensions of the nanosheet fixed. This relaxation was performed using the PBEsol functional. Ionic relaxation was deemed converged when the forces on all atoms were less than 0.01 eV/Å (1.0 x

10-3 Ry/Bohr) and the total energy change was less than 1.0 x 10^{-4} Ry. The relaxation procedure utilized the BFGS algorithm and was completed in 2 BFGS steps after achieving convergence in 3 SCF cycles.

Electronic Structure Calculations: Self-consistent field (SCF) calculations were performed using the PBEsol functional with a plane-wave basis set kinetic energy cutoff of 60 Ry and a k-point grid of 12 x 12 x 3. To accurately capture the electronic structure, we included spin-orbit coupling effects in the SCF calculations. SCF convergence was achieved when the total energy change was less than 1.0 x 10^{-6} Ry.

Band Structure Calculation: Finally, we calculated the electronic band structure of the material, which provides insights into its electronic properties, such as its conductivity and optical properties. This involved determining the allowed energy levels for electrons within the material. To achieve this, we sampled a specific path through the Brillouin zone, which represents the momentum space of the electrons. This multi-step computational approach allowed us to accurately determine the electronic properties of the nanosheet material, starting from an experimentally determined structure and culminating in the calculation of its electronic band structure.

While hybrid functionals and more sophisticated methods like DMFT can provide a higher level of accuracy, our chosen approach of employing the PBEsol functional with carefully chosen cutoff energies and k-point grids, incorporating relativistic effects, and including spin-orbit coupling, offers a reliable description of the electronic structure. This is supported by the observation that our calculations reproduce the band closing behavior at the Γ point, similar to previous DMFT calculations on T'-type RuO_2 using the Abinit package.[2] Furthermore, considering the limitations of our current model, which does not account for defects and other complexities present in real materials, we believe that our chosen level of theory is sufficient for this study.

S6. Optical spectra.

Optical spectra of the colloidal nanosheets were obtained using UV-vis spectrophotometer (Shimadzu, UV-2600i,). The concentration of colloidal nanosheets was 0.01 g L^{-1}. The spectra were obtained by a transmittance mode.

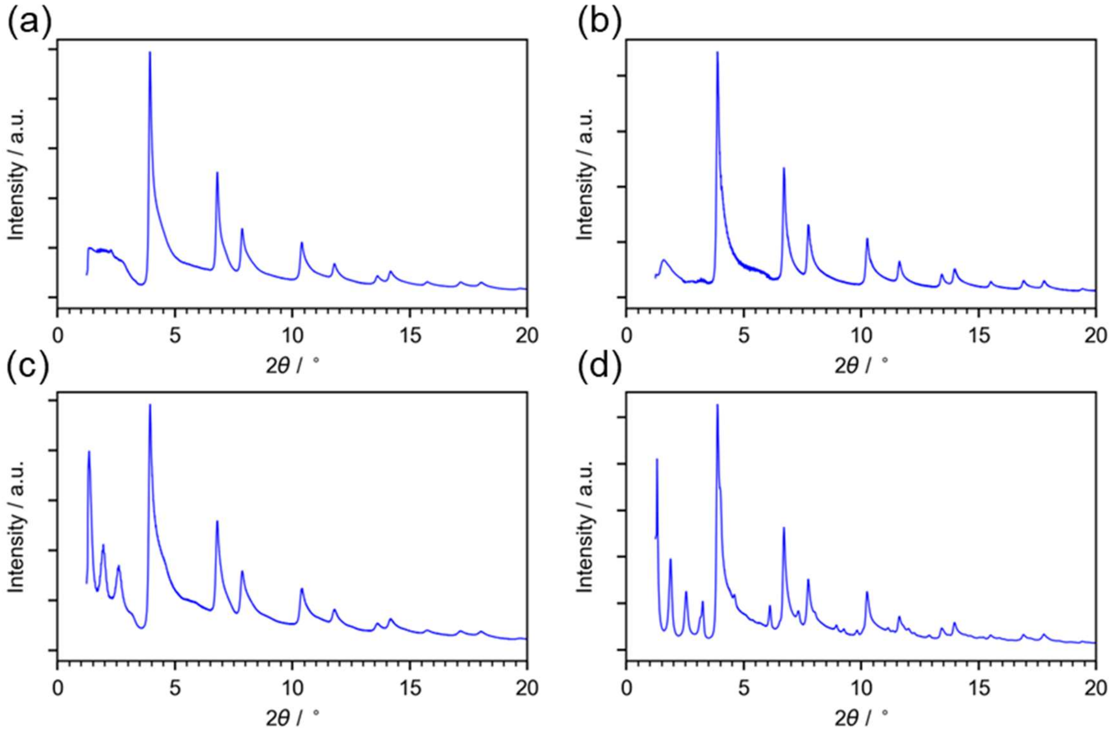

Figure S1 Total scattering patterns in 2θ (λ = 0.181050 Å) for two-dimensional platinum dioxide (a, c) and iridium dioxide (b, d). (a, b) Data for the wet samples. The data were treated by subtracting intensities obtained for the supernatant collected in the centrifuge processes. (c, d) Data for the dried samples. The platinate sample exhibits Bragg peaks at 7.70, 5.33, 3.98, and 3.33 degrees, which correspond to 002, 003, 004 and 005 indices for the d-spacing of ca. 16.0 Å. Likewise, the iridate sample exhibits peaks at 7.93, 5.50, 4.07 and 3.19 degrees, which correspond to 002, 003, 004, and 005 indices for the d-spacing of ca. 16.0 Å.

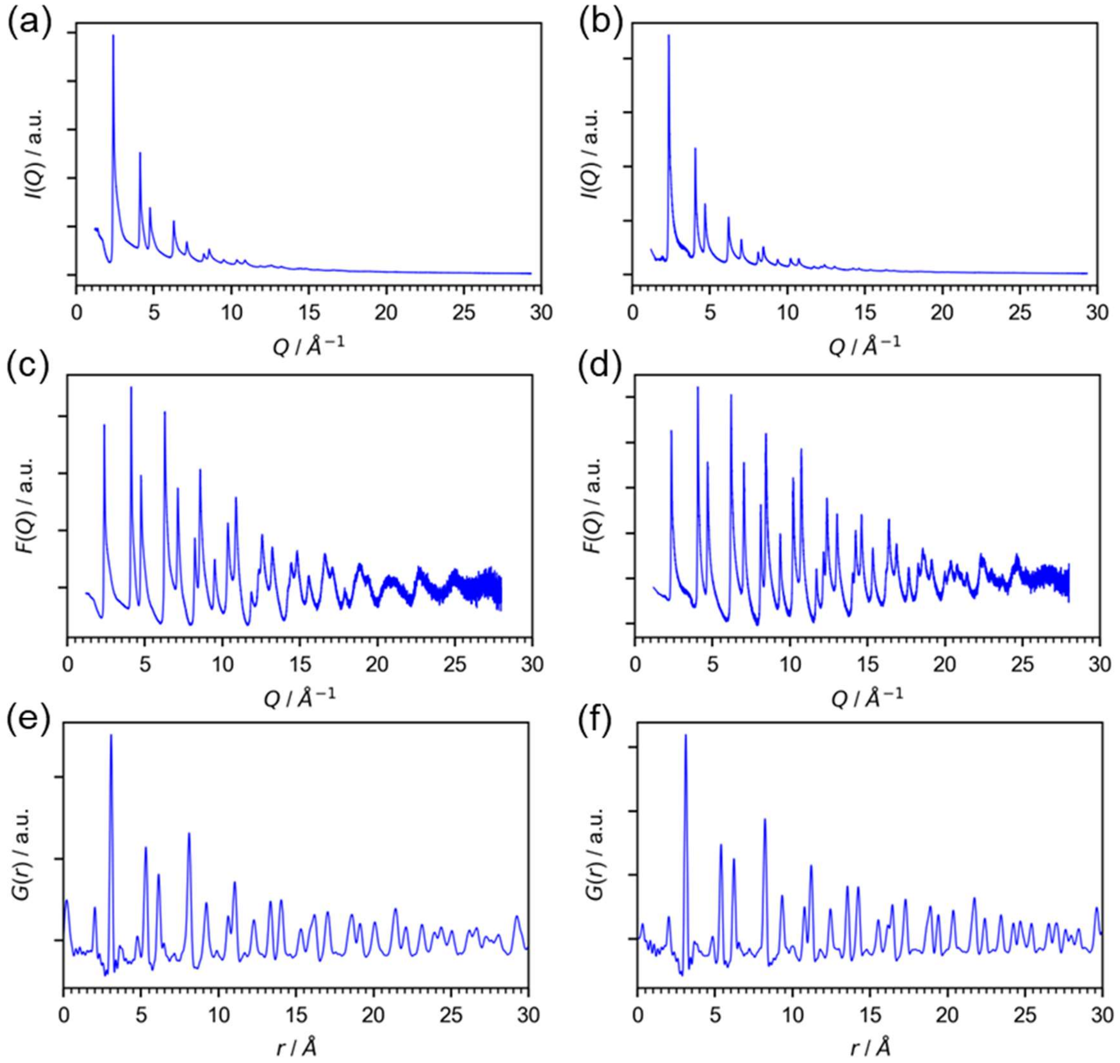

Figure S2 Total scattering data of two-dimensional platinum dioxide (a, c, e) and iridium dioxide (b, d, f). The samples are in wet forms. (a, b) Total scattering intensity vs. amplitude of scattering vector Q. (c, d) Reduced structure function, $F(Q)$. (e, f) Reduced pair distribution functions, $G(r)$.

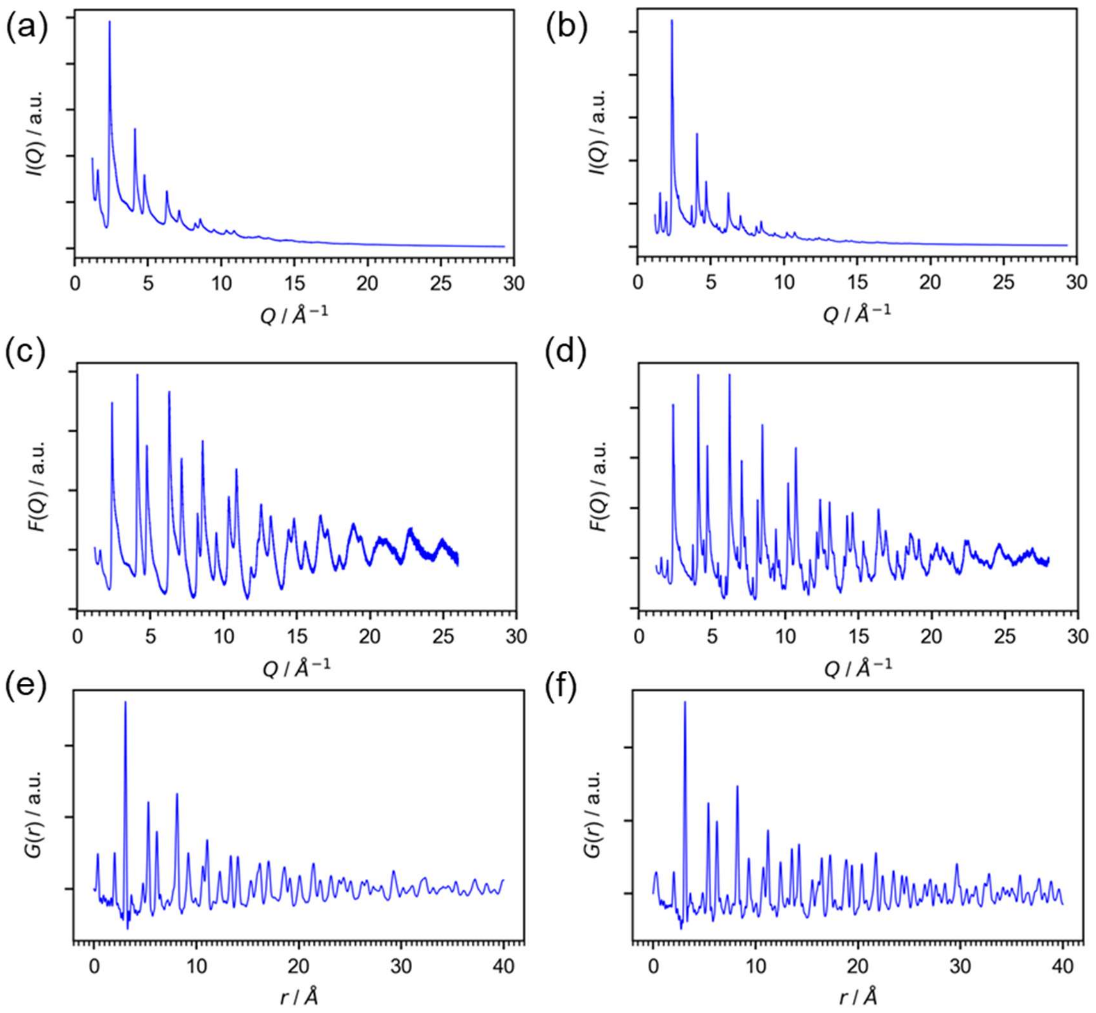

Figure S3 Total scattering data of two-dimensional platinum dioxide (a, c, e) and iridium dioxide (b, d, f). The samples are in dried powder. (a, b) Total scattering intensity vs. amplitude of scattering vector Q. (c, d) Reduced structure function, $F(Q)$. (e, f) Reduced pair distribution functions, $G(r)$.

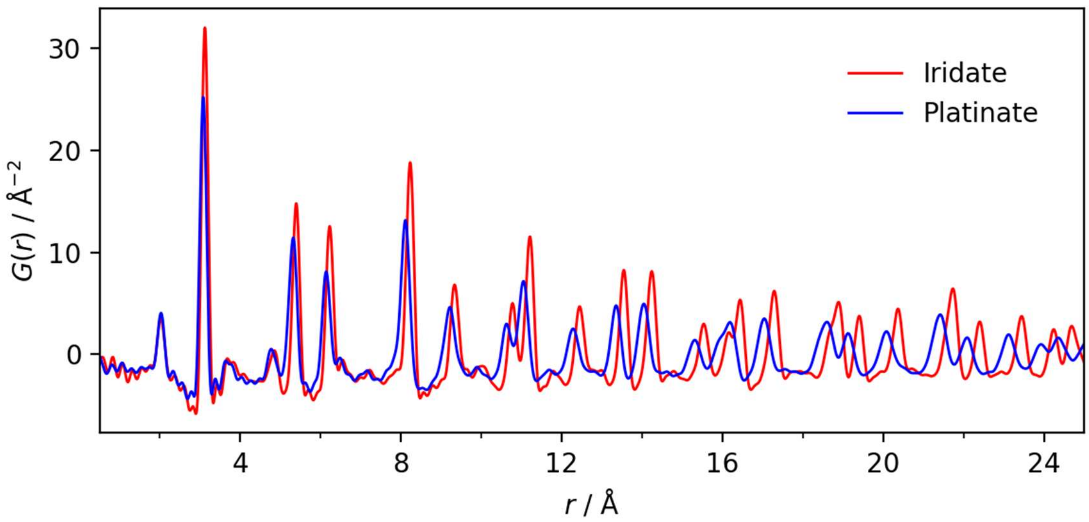

Figure S4 Comparison of reduced pair distribution functions, $G(r)$, of two-dimensional platinum dioxide (platinate) and iridium dioxide (iridate). The samples are in wet forms.

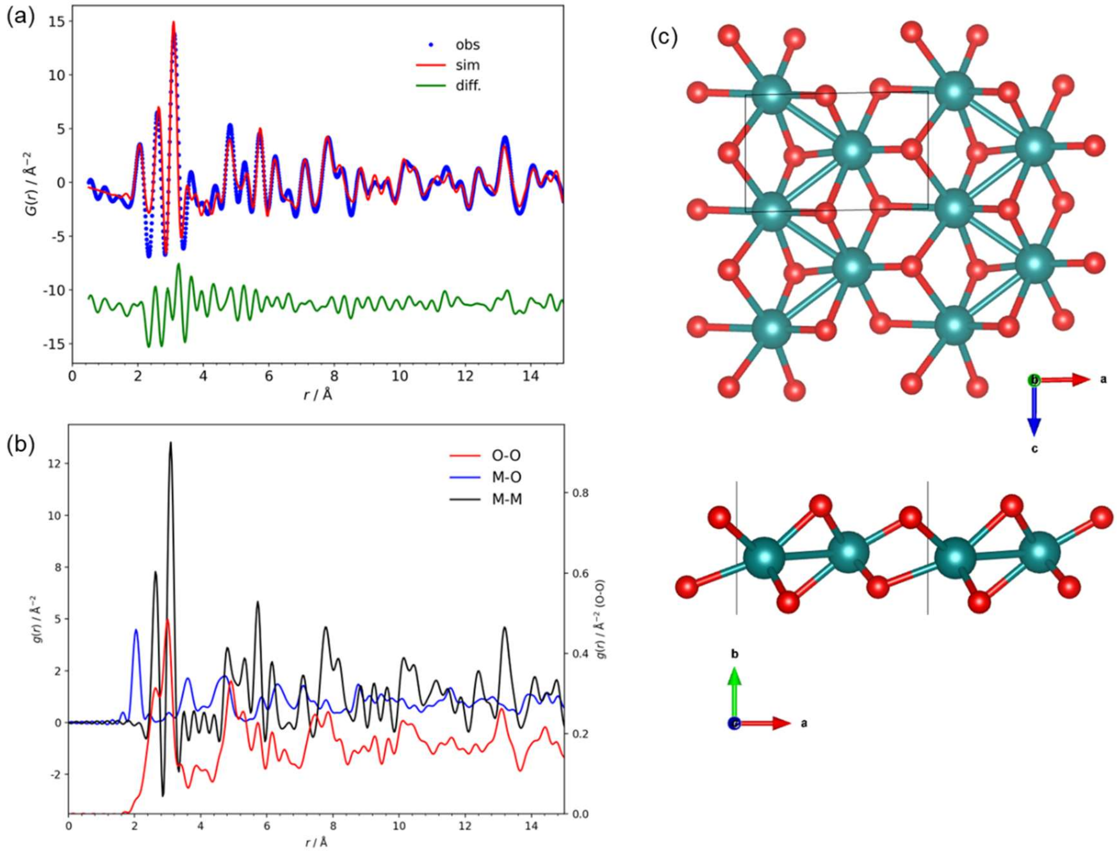

Figure S5. PDF analysis of the previously reported ruthenate nanosheet (Ref. 6). (a) The experimental reduced pair distribution function, G(r), is fitted using the T'-MoS_2-type structure determined in our prior work as the initial model. (b) Calculated partial PDFs for the Ru-Ru, Ru-O, and O-O pairs corresponding to the refined structural model. (c) The T'-MoS_2-type structural model of the ruthenate nanosheet, highlighting the characteristic zigzag chains of short Ru-Ru distances. The refined model shows good agreement with the data and yields a monoclinic unit cell with the following lattice parameters: a = 4.815(6) Å, b = 14.61236(8) Å, c = 3.084(5) Å, and β = 91.1(2)°. The data and model are presented here to support the comparison made in the main text.

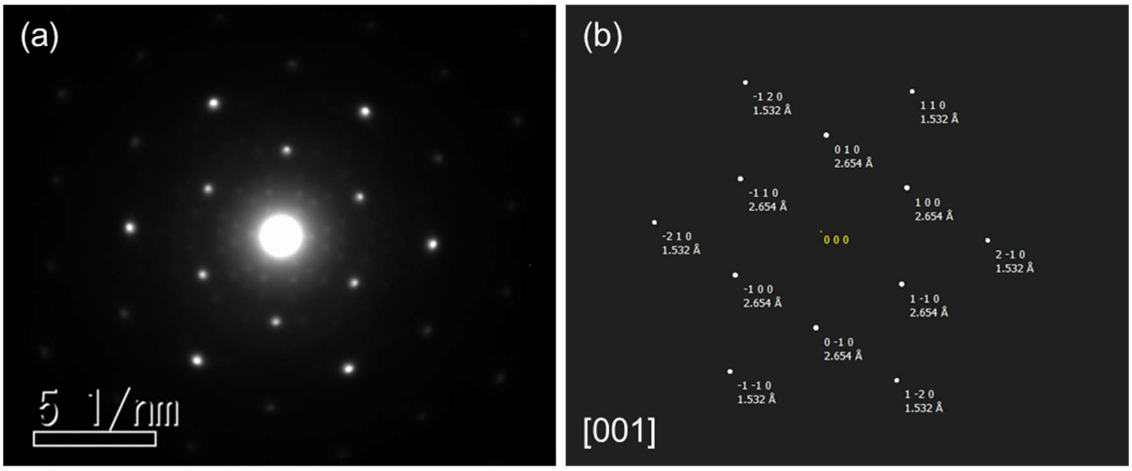

Figure S6 In-plane symmetry analysis of a platinate nanosheet.(a) Experimentally obtained selected-area electron diffraction (SAED) pattern. (b) Simulated electron diffraction pattern for the PtO_2 nanosheet with the T-MoS_2 structure, refined using the pair distribution function (PDF) fitting process. The d-spacing for the observed 100 spot was calculated as 2.8 Å, which corresponds closely to the simulated value of 2.7 Å (falling within the experimental error). The simulation was performed using the ReciPro program,[6] taking into account the kinematical effect. The diffraction spots were simulated using Gaussian functions.

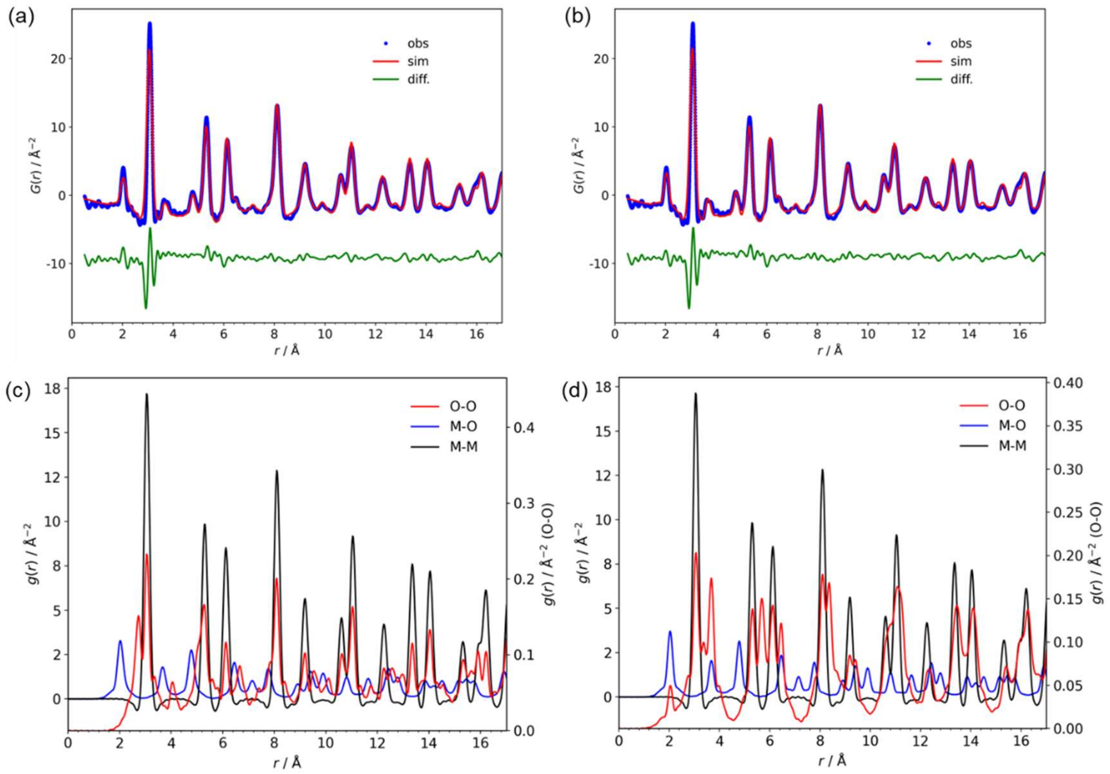

Figure S7. Comparison of PDF fitting results for 2D platinum dioxide using trigonal and hexagonal structure models. The data were collected on a wet powder sample. (a) Fit to the experimental reduced pair distribution function, G(r), using the trigonal model. (b) Fit to G(r) using the hexagonal model. (c) Calculated partial PDFs for the M-M, M-O, and O-O pairs corresponding to the trigonal model. (d) Calculated partial PDFs corresponding to the hexagonal model. Due to its low intensity, the O-O partial PDF is plotted on the secondary y-axis (right). Notably, the hexagonal model requires a physically unrealistic short nearest-neighbor O-O distance of ~2 Å, which is significantly shorter than the ~3 Å typically observed in oxides. This discrepancy supports the conclusion that the trigonal model is the more chemically reasonable structure.

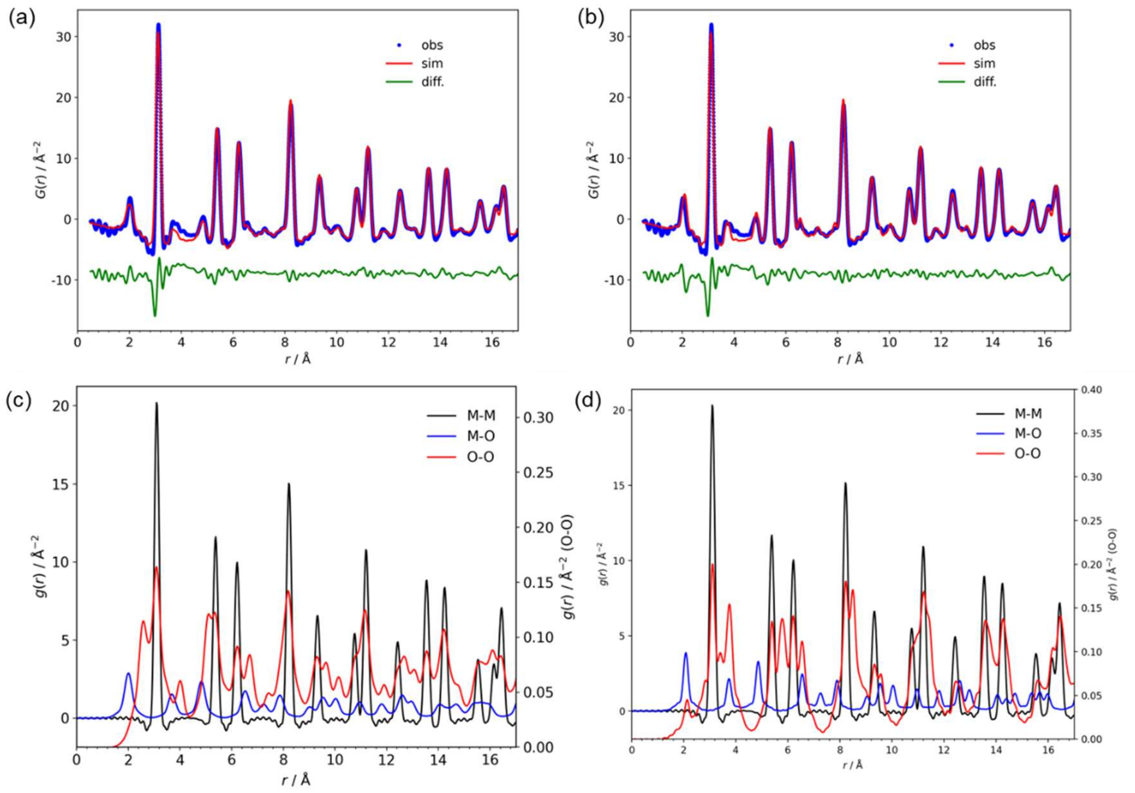

Figure S8. Comparison of PDF fitting results for 2D iridium dioxide using trigonal and hexagonal structure models. The data were collected on a wet powder sample. (a) Fit to the experimental reduced pair distribution function, G(r), using the trigonal model. (b) Fit to G(r) using the hexagonal model. (c) Calculated partial PDFs for the M-M, M-O, and O-O pairs corresponding to the trigonal model. (d) Calculated partial PDFs corresponding to the hexagonal model. Due to its low intensity, the O-O partial PDF is plotted on the secondary y-axis (right). As with the platinate case, the hexagonal model requires a physically unrealistic short nearest-neighbor O-O distance of ~2 Å, significantly shorter than the ~3 Å typically observed in oxides. This discrepancy supports the conclusion that the trigonal model is the more chemically reasonable structure for the iridate nanosheets as well.

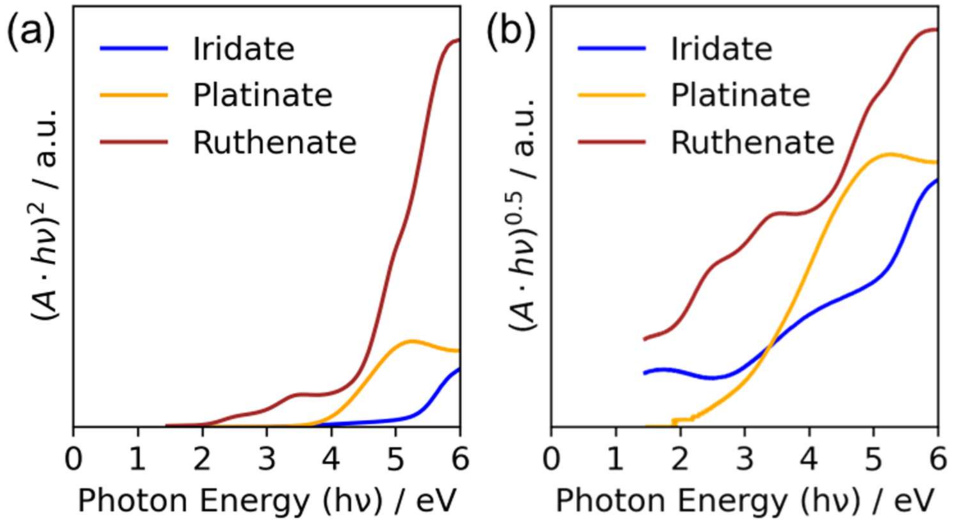

Figure S9 Tauc analysis for estimating the optical band gaps of the PMD-nanosheets. The absorption data are plotted according to the Tauc model for (a) direct transitions ($(Ah\nu)^2$ vs. hν) and (b) indirect transitions ($(Ah\nu)^{0.5}$ vs. hν).

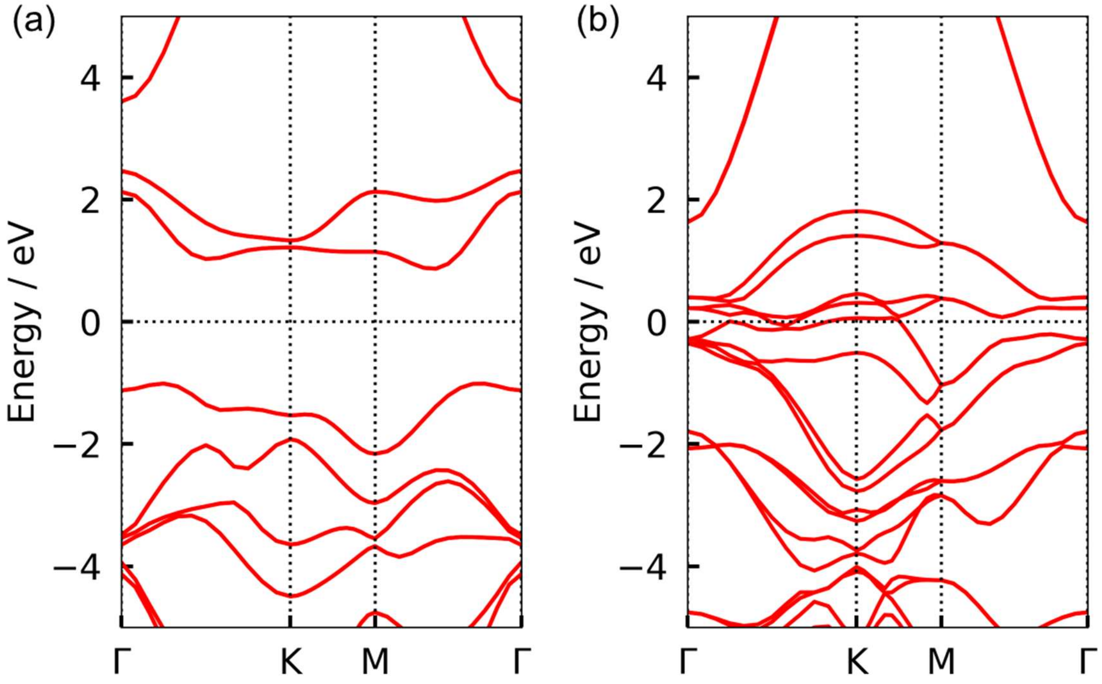

Figure S10 Band structures of T-type PtO_2 (a) and H-type PtO_2 (b) calculated using density functional theory (DFT) with the projector augmented wave (PAW) method and the PBEsol exchange-correlation functional as implemented in Quantum ESPRESSO. The Fermi level is set to 0 eV. High-symmetry points Γ, K, and M in the Brillouin zone are shown, corresponding to the in-plane directions of the 2D materials.

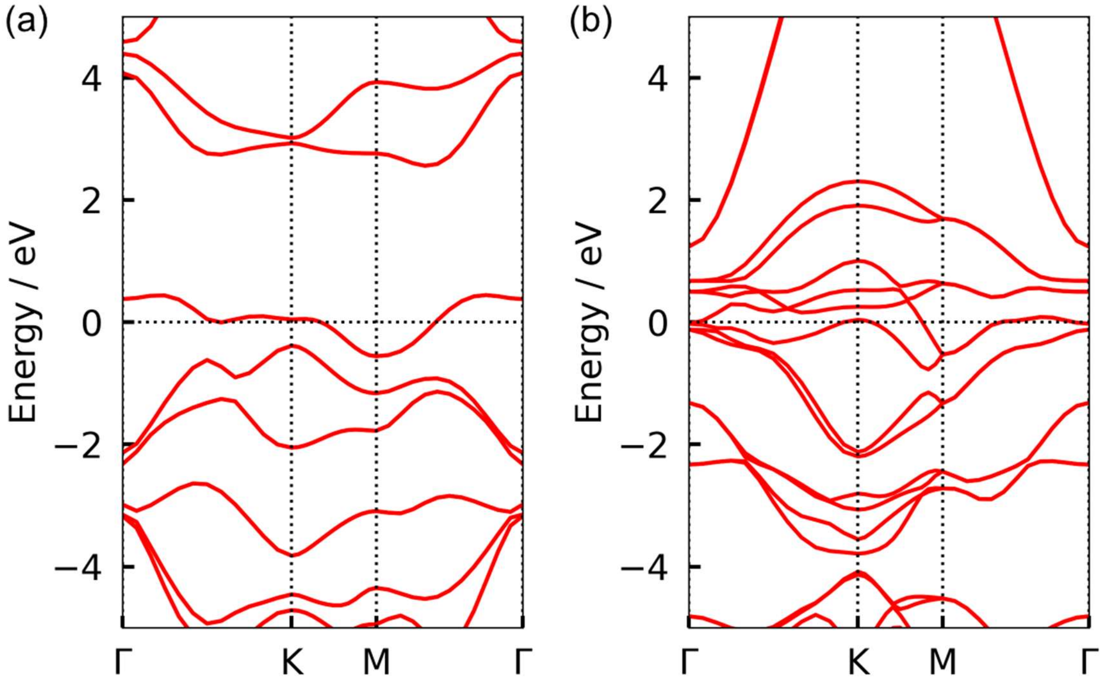

Figure S11 Band structures of T-type IrO_2 (a) and H-type IrO_2 (b) calculated using density functional theory (DFT) with the projector augmented wave (PAW) method and the PBEsol exchange-correlation functional as implemented in Quantum ESPRESSO. The Fermi level is set to 0 eV. High-symmetry points Γ, K, and M in the Brillouin zone are shown, corresponding to the in-plane directions of the 2D materials.

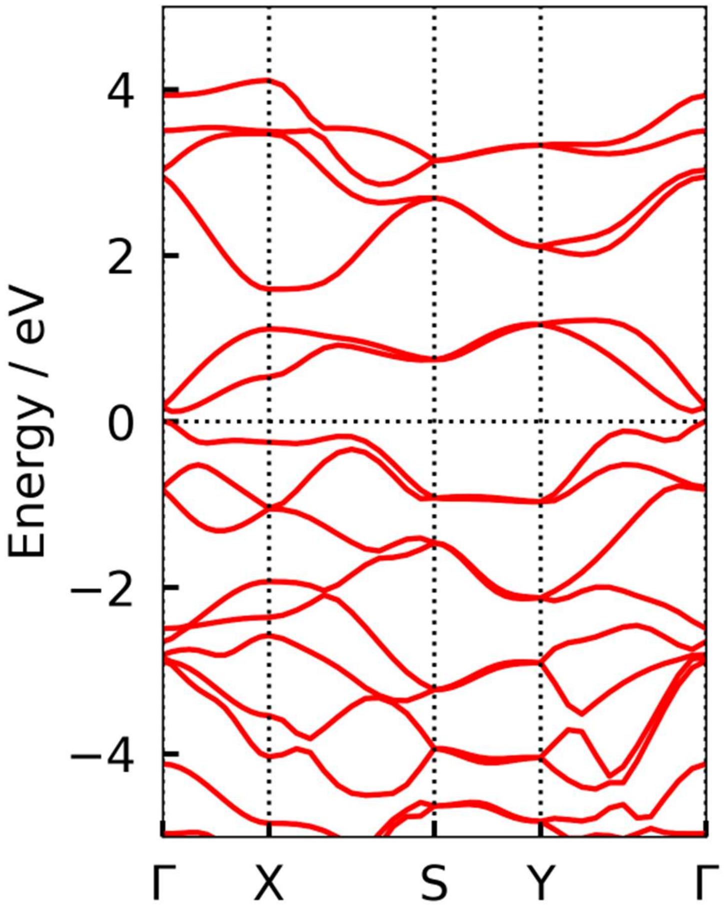

Figure S12 Band structures of T'-type RuO_2 calculated using density functional theory (DFT) with the projector augmented wave (PAW) method and the PBEsol exchange-correlation functional as implemented in Quantum ESPRESSO. The Fermi level is set to 0 eV. High-symmetry points Γ, X, S and Y in the Brillouin zone are shown, corresponding to the in-plane directions of the 2D materials.

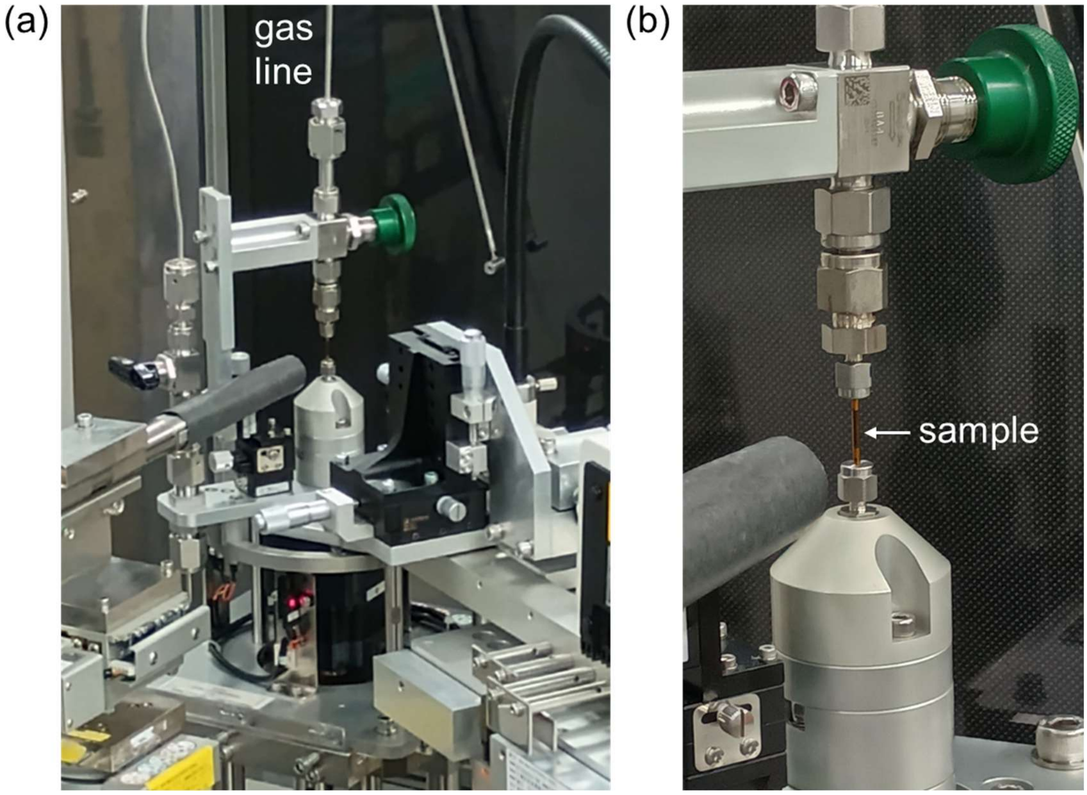

Figure S13 Experimental setup used for the in-situ PDF measurements. The sample capillary was initially evacuated to a gauge pressure of -0.096 MPa. Subsequently, hydrogen gas was introduced, adjusting the pressure inside the capillary to either approximately 0.0 MPa (atmospheric pressure) or -0.092 MPa gauge for the measurements.

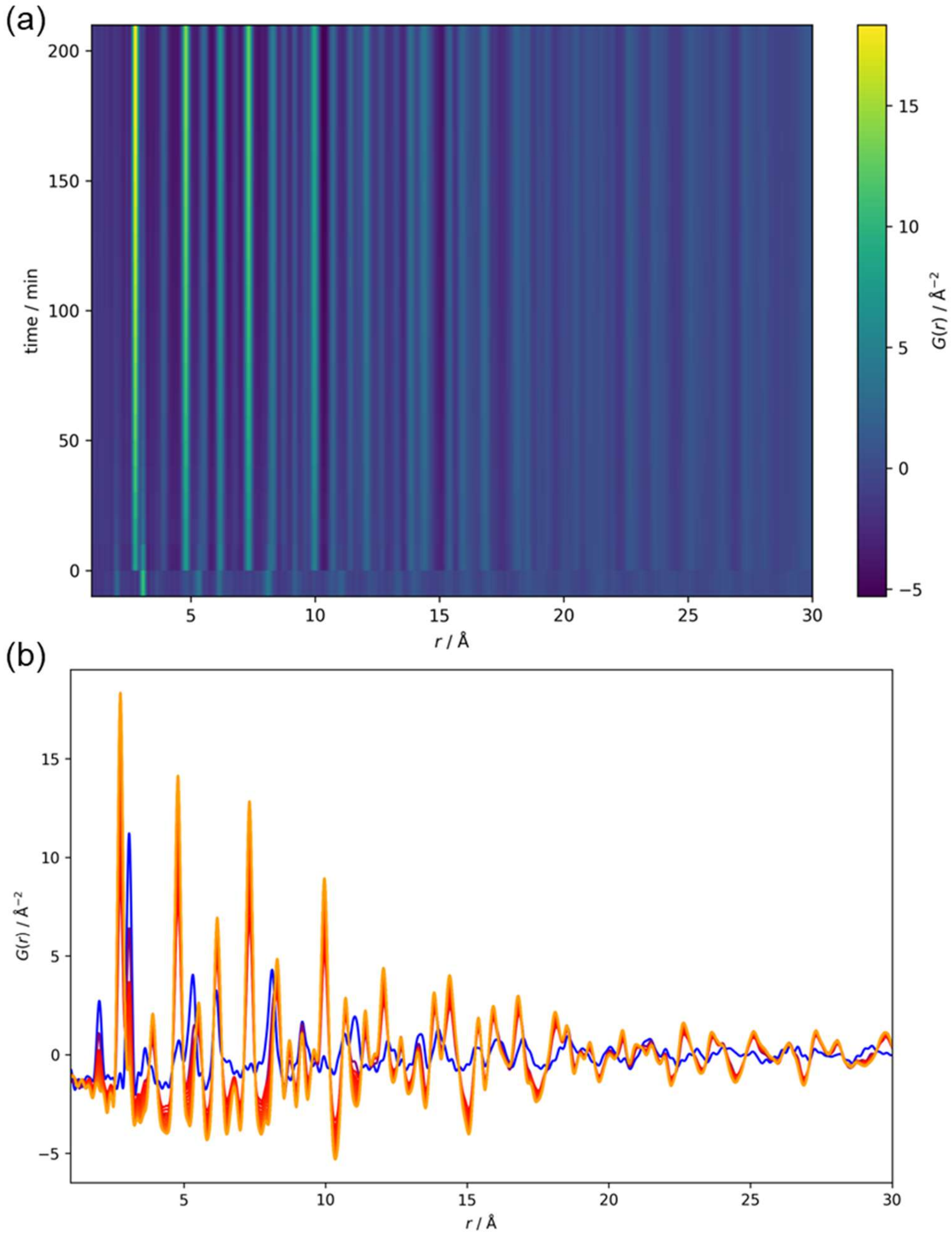

Figure S14 In-situ PDF data for platinate nanosheets during reduction with H_2 gas (0.0 MPa gauge pressure), showing an extended r-range. This dataset corresponds to the experiment detailed in Figure 4 of the main text. (a) Time-resolved 2D contour plot of $G(r)$ upon H_2 introduction (t=0). (b) Selected PDF patterns G(r) illustrating various stages: initial state under vacuum (blue), during the transition to H_2 atmosphere (purple), and under H_2 atmosphere (red/orange hues). Data are shown up to r = 30 Å.

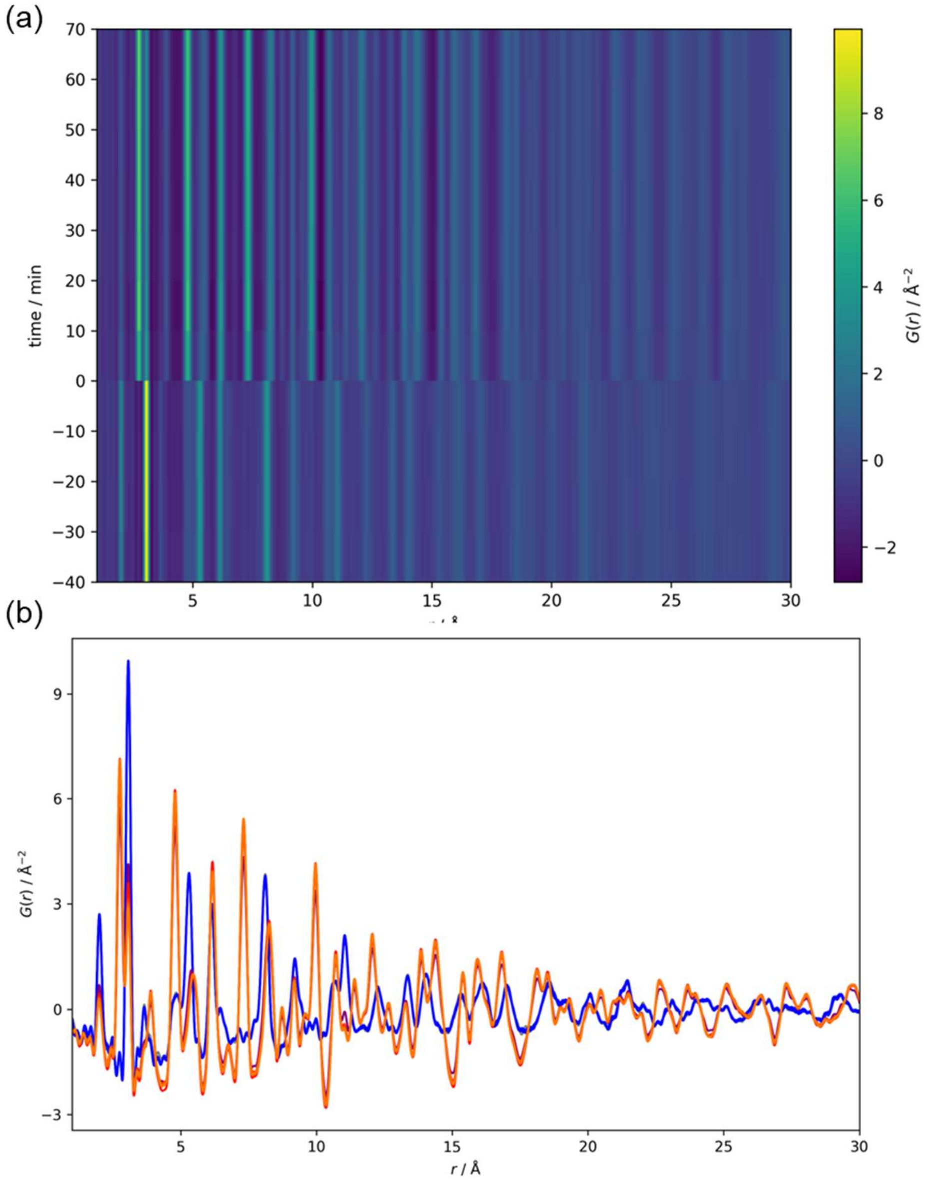

Figure S15 In-situ PDF data for platinate nanosheets under various atmospheric conditions, followed by reduction with H_2 gas at -0.092 MPa gauge pressure. (a) Time-resolved 2D contour plot of $G(r)$ during H_2 exposure (t=0 marks H_2 introduction). (b) Selected PDF patterns $G(r)$ at different stages. Prior to H_2 exposure, the sample was characterized by a 10-minute measurement in air, followed by three consecutive 10-minute measurements under vacuum to confirm no structural changes occurred due to evacuation alone. The subsequent data shows the reduction process under the H_2 atmosphere. Data are shown up to $r = 30$ Å.

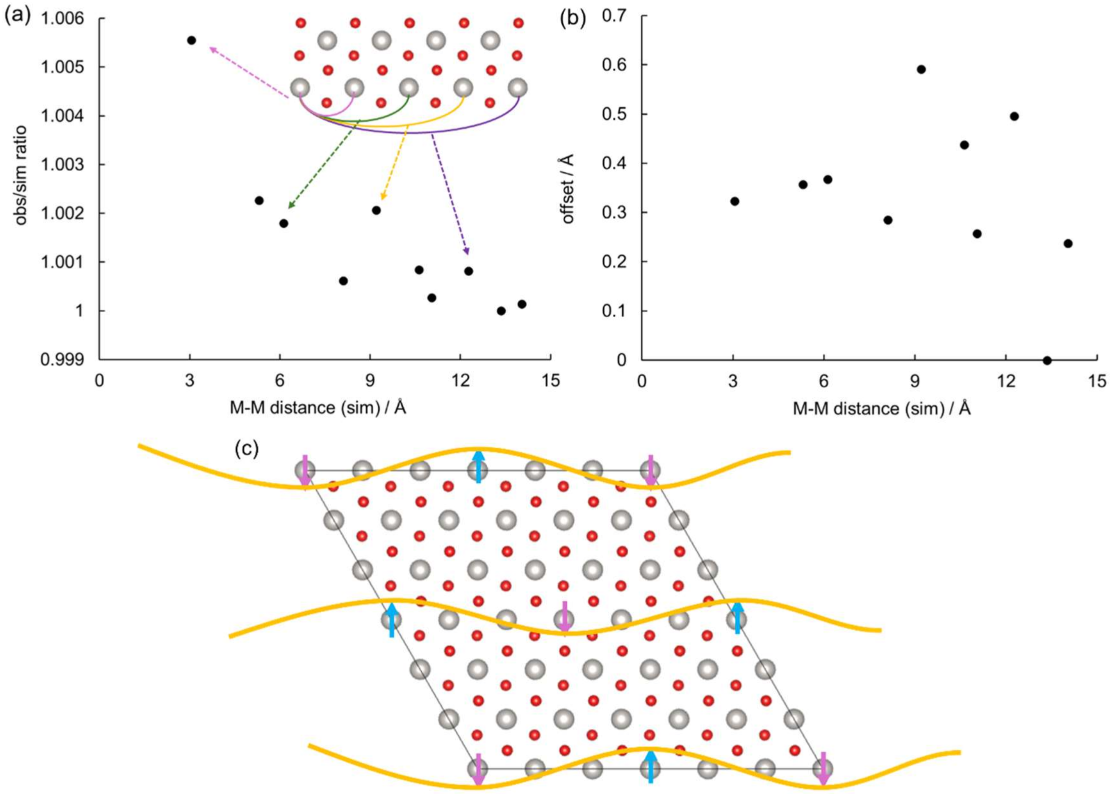

Figure S16 Analysis of the discrepancy between observed and simulated pair distribution functions (PDFs). (a) The ratio of observed to simulated M-M distances plotted as a function of the M-M distance in the ideal T-type structure model. (b) Calculated vertical offset values (Δz), derived under the assumption that the bond length discrepancies originate solely from out-of-plane displacements (buckling) of metal ions. (c) Proposed structural interpretation of the offsets. The yellow wavy curves illustrate the sinusoidal modulation along the unit cell axes. Blue and red arrows indicate upward and downward displacements of the metal ions, respectively. The largest offset, observed at an M-M distance of ~9.2 Å, corresponds to the half-wavelength (peak-to-valley distance) of the modulation, which equals three times the unit cell parameter of the T-type structure model. This suggests a full modulation periodicity of 6*a*, indicating that a 6 × 6 supercell is required to describe this commensurate sinusoidal buckling.

Table S1 Two-dimensional X-ray diffraction information of T-type PtO_2 model obtained by PDF fitting. Diffraction information including hkl indices, d spacing, and intensities was simulated using the VESTA program and those for in-plane diffraction was listed. This pattern can account for the experimental data reported by Takimoto et al.[7]

hkl	d / Å	1/d / $Å^{-1}$	Relative intensity
100	2.665	0.375	100
110	1.539	0.650	28.1
200	1.332	0.751	11.2
210	1.007	0.993	10.2

Structure information obtained by the PDF fitting (in cif format)

```
data_2d-platinate_trigonal.cif
_audit_creation_method    Orochi program
_cell_length_a    3.06578(4)
_cell_length_b    3.06578(4)
_cell_length_c    7.70000
_cell_angle_alpha    90.00000
_cell_angle_beta    90.00000
_cell_angle_gamma    120.00000
_symmetry_space_group_name_H-M    P-3m1
_symmetry_Int_Tables_number    164

loop_
_atom_site_type_symbol
_atom_site_label
_atom_site_fract_x
_atom_site_fract_y
_atom_site_fract_z
_atom_site_U_iso_or_equiv
_atom_site_occupancy
Pt    Pt1    0.00000    0.00000    0.50000    0.00285    1.00000
O    O1    0.66667    0.33333    0.62739(19)    0.00476    1.00000
```

```
data_2d-iridate_trigonal.cif
_audit_creation_date    2025-01-09
_audit_creation_method    Orochi program
_cell_length_a    3.10756
_cell_length_b    3.10756
_cell_length_c    7.70000
_cell_angle_alpha    90.00000
_cell_angle_beta    90.00000
_cell_angle_gamma    120.00000
_symmetry_space_group_name_H-M    P-3m1
_symmetry_Int_Tables_number    164

loop_
_atom_site_type_symbol
_atom_site_label
_atom_site_fract_x
_atom_site_fract_y
_atom_site_fract_z
_atom_site_U_iso_or_equiv
_atom_site_occupancy
Ir    Ir1    0.00000    0.00000    0.50000    0.00286    1.00000
O    O1    0.66667    0.33333    0.6203(3)    0.00657    1.00000
```

```
data_2d-platinate_hexagonal.cif
_audit_creation_method    Orochi program
_cell_length_a    3.06507(3)
_cell_length_b    3.06507(3)
_cell_length_c    7.70000
_cell_angle_alpha    90.00000
_cell_angle_beta    90.00000
_cell_angle_gamma    120.00000
_symmetry_space_group_name_H-M    P-6m2
_symmetry_Int_Tables_number    187

loop_
_atom_site_type_symbol
_atom_site_label
_atom_site_fract_x
_atom_site_fract_y
_atom_site_fract_z
_atom_site_U_iso_or_equiv
_atom_site_occupancy
Pt    Pt1    0.33333    0.66667    0.50000    0.00288    1.00000
O    O1    0.00000    0.00000    0.6284(3)    0.00492    1.00000
```

```
data_2d-iridate_hexagonal.cif
_audit_creation_method    Orochi program
_cell_length_a    3.10743(4)
_cell_length_b    3.10743(4)
_cell_length_c    7.70000
_cell_angle_alpha    90.00000
_cell_angle_beta    90.00000
_cell_angle_gamma    120.00000
_symmetry_space_group_name_H-M    P-6m2
_symmetry_Int_Tables_number    187

loop_
_atom_site_type_symbol
_atom_site_label
_atom_site_fract_x
_atom_site_fract_y
_atom_site_fract_z
_atom_site_U_iso_or_equiv
_atom_site_occupancy
Ir    Ir1    0.33333    0.66667    0.50000    0.00277    1.00000
O    O1    0.00000    0.00000    0.6216(3)    0.00649    1.00000
```

```
data_2d-ruthenate_T'.cif
_audit_creation_method    Orochi program
_cell_length_a    4.815(6)
_cell_length_b    14.61236(8)
_cell_length_c    3.084(5)
_cell_angle_alpha    90.00000
_cell_angle_beta    91.1(2)
_cell_angle_gamma    90.00000
_symmetry_space_group_name_H-M    P1
_symmetry_Int_Tables_number    1
_symmetry_cell_setting    triclinic

loop_
_atom_site_type_symbol
_atom_site_label
_atom_site_fract_x
_atom_site_fract_y
_atom_site_fract_z
_atom_site_U_iso_or_equiv
_atom_site_occupancy
Ru    Ru1    0.585(5)    0.22(2)    0.488(8)    0.0029(6)    1.00000
Ru    Ru2    0.145(3)    0.21(2)    0.0007(11)    0.00431(5)    1.00000
O    O1    0.91(5)    0.28(4)    0.49(10)    0.0169(14)    1.00000
O    O4    0.27(4)    0.134(13)    0.53(12)    0.00475(14)    1.00000
O    O2    0.44(4)    0.30(3)    1.02(16)    0.00190(5)    1.00000
O    O3    0.74(5)    0.16(4)    0.97(6)    0.00182(18)    1.00000
```

data_2d-platinate_6x6_modulated.cif
_cell_length_a 18.377(4)
_cell_length_b 18.37683
_cell_length_c 15.0001(2)
_cell_angle_alpha 90.00000
_cell_angle_beta 90.00000
_cell_angle_gamma 120.00000
_symmetry_space_group_name_H-M P1
_symmetry_Int_Tables_number 1
_symmetry_cell_setting hexagonal

loop_
_atom_site_type_symbol
_atom_site_label
_atom_site_fract_x
_atom_site_fract_y
_atom_site_fract_z
_atom_site_U_iso_or_equiv
_atom_site_occupancy
Pt Pt1 0.00000 0.00000 0.53398 0(18) 1.00000
Pt Pt1 0.00000 0.16667 0.51699 0.00207 1.00000
Pt Pt1 0.00000 0.33333 0.48301 0.00207 1.00000
Pt Pt1 0.00000 0.50000 0.46602 0.00207 1.00000
Pt Pt1 0.00000 0.66667 0.48301 0.00207 1.00000
Pt Pt1 0.00000 0.83333 0.51699 0.00207 1.00000
Pt Pt1 0.16667 0.00000 0.51699 0.00207 1.00000
Pt Pt1 0.16667 0.16667 0.50849 0.00207 1.00000
Pt Pt1 0.16667 0.33333 0.49151 0.00207 1.00000
Pt Pt1 0.16667 0.50000 0.48301 0.00207 1.00000
Pt Pt1 0.16667 0.66667 0.49151 0.00207 1.00000
Pt Pt1 0.16667 0.83333 0.50849 0.00207 1.00000
Pt Pt1 0.33333 0.00000 0.48301 0.00207 1.00000
Pt Pt1 0.33333 0.16667 0.49151 0.00207 1.00000
Pt Pt1 0.33333 0.33333 0.50849 0.00207 1.00000
Pt Pt1 0.33333 0.50000 0.51699 0.00207 1.00000
Pt Pt1 0.33333 0.66667 0.50849 0.00207 1.00000

Pt	Pt1	0.33333	0.83333	0.49151	0.00207	1.00000
Pt	Pt1	0.50000	0.00000	0.46602	0.00207	1.00000
Pt	Pt1	0.50000	0.16667	0.48301	0.00207	1.00000
Pt	Pt1	0.50000	0.33333	0.51699	0.00207	1.00000
Pt	Pt1	0.50000	0.50000	0.53398	0.00207	1.00000
Pt	Pt1	0.50000	0.66667	0.51699	0.00207	1.00000
Pt	Pt1	0.50000	0.83333	0.48301	0.00207	1.00000
Pt	Pt1	0.66667	0.00000	0.48301	0.00207	1.00000
Pt	Pt1	0.66667	0.16667	0.49151	0.00207	1.00000
Pt	Pt1	0.66667	0.33333	0.50849	0.00207	1.00000
Pt	Pt1	0.66667	0.50000	0.51699	0.00207	1.00000
Pt	Pt1	0.66667	0.66667	0.50849	0.00207	1.00000
Pt	Pt1	0.66667	0.83333	0.49151	0.00207	1.00000
Pt	Pt1	0.83333	0.00000	0.51699	0.00207	1.00000
Pt	Pt1	0.83333	0.16667	0.50849	0.00207	1.00000
Pt	Pt1	0.83333	0.33333	0.49151	0.00207	1.00000
Pt	Pt1	0.83333	0.50000	0.48301	0.00207	1.00000
Pt	Pt1	0.83333	0.66667	0.49151	0.00207	1.00000
Pt	Pt1	0.83333	0.83333	0.50849	0.00207	1.00000
O	O1	0.117(4)	0.060(4)	0.5913(2)	0.0(5)	1.00000
O	O1	0.118(6)	0.2299(5)	0.5713(4)	0.00203	1.00000
O	O1	0.118(11)	0.3993(2)	0.5478(5)	0.00203	1.00000
O	O1	0.117(8)	0.5602(10)	0.5446(10)	0.00203	1.00000
O	O1	0.117(10)	0.7299(7)	0.565(17)	0.00203	1.00000
O	O1	0.118(3)	0.8895(11)	0.5871(6)	0.00203	1.00000
O	O1	0.2828(5)	0.06(8)	0.5617(5)	0.00203	1.00000
O	O1	0.283(9)	0.230(12)	0.5673(10)	0.00203	1.00000
O	O1	0.2826(2)	0.3997(3)	0.5735(6)	0.00203	1.00000
O	O1	0.283(17)	0.5604(10)	0.5746(5)	0.00203	1.00000
O	O1	0.283(17)	0.7303(3)	0.5688(8)	0.00203	1.00000
O	O1	0.2823(4)	0.8896(7)	0.5630(13)	0.00203	1.00000
O	O1	0.4497(4)	0.060(4)	0.5383(8)	0.00203	1.00000
O	O1	0.450(13)	0.2295(5)	0.5639(8)	0.00203	1.00000
O	O1	0.450(17)	0.3999(15)	0.5940(14)	0.00203	1.00000
O	O1	0.4498(6)	0.55981(3)	0.5978(8)	0.00203	1.00000
O	O1	0.449(18)	0.7303(10)	0.5720(11)	0.00203	1.00000

O	O1	0.4495(5)	0.8903(10)	0.5432(4)	0.00203	1.00000
O	O1	0.6168(7)	0.059(7)	0.5446(11)	0.00203	1.00000
O	O1	0.6172(3)	0.229(9)	0.565(19)	0.00203	1.00000
O	O1	0.6170(7)	0.3996(3)	0.588(9)	0.00203	1.00000
O	O1	0.6167(4)	0.5597(13)	0.5913(6)	0.00203	1.00000
O	O1	0.617(18)	0.7293(13)	0.5714(10)	0.00203	1.00000
O	O1	0.6166(8)	0.8904(2)	0.5486(3)	0.00203	1.00000
O	O1	0.7826(6)	0.060(6)	0.5744(4)	0.00203	1.00000
O	O1	0.7826(5)	0.2294(3)	0.5691(3)	0.00203	1.00000
O	O1	0.7831(7)	0.3996(5)	0.5626(6)	0.00203	1.00000
O	O1	0.78317(2)	0.5603(5)	0.562(16)	0.00203	1.00000
O	O1	0.7832(11)	0.7297(8)	0.567(14)	0.00203	1.00000
O	O1	0.7827(3)	0.890(18)	0.5737(2)	0.00203	1.00000
O	O1	0.9498(6)	0.061(8)	0.5979(4)	0.00203	1.00000
O	O1	0.9502(9)	0.2301(4)	0.572(10)	0.00203	1.00000
O	O1	0.9503(7)	0.399(12)	0.5421(7)	0.00203	1.00000
O	O1	0.9496(7)	0.5602(2)	0.5383(7)	0.00203	1.00000
O	O1	0.9501(6)	0.7298(3)	0.5641(4)	0.00203	1.00000
O	O1	0.9497(4)	0.8902(3)	0.5931(11)	0.00203	1.00000
O	O2	0.052(9)	0.113(4)	0.4566(5)	0.00203	1.00000
O	O2	0.052(6)	0.280(15)	0.4261(5)	0.00203	1.00000
O	O2	0.05(19)	0.447(18)	0.4016(2)	0.00203	1.00000
O	O2	0.050(16)	0.6133(11)	0.4073(13)	0.00203	1.00000
O	O2	0.05(17)	0.7803(11)	0.4381(4)	0.00203	1.00000
O	O2	0.050(8)	0.9472(9)	0.4625(5)	0.00203	1.00000
O	O2	0.220(15)	0.112(4)	0.4368(2)	0.00203	1.00000
O	O2	0.2204(2)	0.2795(3)	0.4309(4)	0.00203	1.00000
O	O2	0.221(15)	0.4464(14)	0.4259(3)	0.00203	1.00000
O	O2	0.220(13)	0.6134(11)	0.427(18)	0.00203	1.00000
O	O2	0.2198(3)	0.7803(5)	0.4329(12)	0.00203	1.00000
O	O2	0.220(16)	0.9472(6)	0.4380(11)	0.00203	1.00000
O	O2	0.3895(3)	0.113(5)	0.4123(2)	0.00203	1.00000
O	O2	0.3901(3)	0.2802(6)	0.4370(14)	0.00203	1.00000
O	O2	0.3900(3)	0.4465(4)	0.457(7)	0.00203	1.00000
O	O2	0.3902(6)	0.6131(9)	0.4515(5)	0.00203	1.00000
O	O2	0.3894(3)	0.7799(2)	0.427(15)	0.00203	1.00000

O	O2	0.3897(4)	0.9471(5)	0.4070(18)	0.00203	1.00000
O	O2	0.5595(11)	0.113(9)	0.408(8)	0.00203	1.00000
O	O2	0.5603(3)	0.2802(2)	0.438(19)	0.00203	1.00000
O	O2	0.5596(16)	0.447(7)	0.4616(7)	0.00203	1.00000
O	O2	0.5594(2)	0.6126(2)	0.4560(4)	0.00203	1.00000
O	O2	0.5594(4)	0.780(11)	0.4260(12)	0.00203	1.00000
O	O2	0.5602(13)	0.9470(5)	0.4022(4)	0.00203	1.00000
O	O2	0.721(13)	0.114(18)	0.4271(9)	0.00203	1.00000
O	O2	0.7204(6)	0.2795(4)	0.4327(3)	0.00203	1.00000
O	O2	0.7204(3)	0.4465(4)	0.4377(6)	0.00203	1.00000
O	O2	0.7197(4)	0.6125(2)	0.4367(9)	0.00203	1.00000
O	O2	0.7204(7)	0.7797(3)	0.4306(3)	0.00203	1.00000
O	O2	0.7202(5)	0.9466(2)	0.4261(3)	0.00203	1.00000
O	O2	0.8896(13)	0.112(11)	0.4517(5)	0.00203	1.00000
O	O2	0.8904(5)	0.2798(5)	0.4271(10)	0.00203	1.00000
O	O2	0.8896(12)	0.4470(5)	0.407(18)	0.00203	1.00000
O	O2	0.8895(4)	0.613(3)	0.4123(11)	0.00203	1.00000
O	O2	0.8902(3)	0.7805(7)	0.4373(10)	0.00203	1.00000
O	O2	0.890(12)	0.9464(3)	0.4564(3)	0.00203	1.00000

Reference:

(1) Tominaka, S.; Yamada, H.; Hiroi, S.; Kawaguchi, S. I.; Ohara, K. Lepidocrocite-Type Titanate Formation from Isostructural Prestructures under Hydrothermal Reactions: Observation by Synchrotron X-Ray Total Scattering Analyses. *ACS Omega* 2018, *3* (8), 8874–8881. https://doi.org/10.1021/acsomega.8b01693.

(2) Tominaka, S.; Tominaka, S.; Ishibiki, R.; Fujino, A.; Kawakami, K.; Ohara, K. Geometrical Frustration of B-H Bonds in Layered Hydrogen Borides Accessible by Soft Chemistry. *Chem* 2020, *6* (2), 406–418. https://doi.org/10.1016/j.chempr.2019.11.006.

(3) Farrow, C. L.; Shi, C.; Peng, X.; Juhas, P.; Billinge, S. J. L. Robust Structure and Morphology Parameters for CdS Nanoparticles by Combining Small-Angle X-Ray Scattering and Atomic Pair Distribution Function Data in a Complex Modeling Framework Research Papers. *J Appl Crystallogr* 2013, *47*, 561–565. https://doi.org/10.1107/S1600576713034055.

(4) Olds, D.; Wang, H.; Page, K. DShaper : An Approach for Handling Missing Low-Q Data in Pair Distribution Function Analysis of Nanostructured Systems Research Papers. *J Appl Crystallogr* 2015, *48*, 1651–1659. https://doi.org/10.1107/S1600576715016581.

(5) Sonobe, K.; Tominaka, S.; Sugimoto, W. Symmetric Breakage-Induced Semimetallic State: Polymorphism in Ruthenate Nanosheets. *J Am Chem Soc* 2022, *144* (33), 15008–15012. https://doi.org/10.1021/jacs.2c05951.

(6) Takimoto, D.; Toma, S.; Suda, Y.; Shirokura, T.; Tokura, Y.; Fukuda, K.; Matsumoto, M.; Imai, H.; Sugimoto, W. Platinum Nanosheets Synthesized via Topotactic Reduction of Single-Layer Platinum Oxide Nanosheets for Electrocatalysis. *Nat Commun* 2023, *14* (1), 19. https://doi.org/10.1038/s41467-022-35616-4.
